# Immunomodulatory Effects of a New Ethynylpiperidine Derivative: Enhancement of CD4^+^FoxP3^+^ Regulatory T Cells in Experimental Acute Lung Injury

**DOI:** 10.3390/biomedicines13123017

**Published:** 2025-12-09

**Authors:** Marina K. Balabekova, Gulgul K. Kairanbayeva, Valentina K. Yu, Symbat Zhumakova, Mariya Li, Tulegen M. Seilkhanov, Khaidar S. Tassibekov, Milana A. Alimova, Meruyert B. Mussilim, Akerke Ardakkyzy Ramazanova

**Affiliations:** 1 Department of Pathological Physiology Named After Professor A.N. Nurmukhambetov, Asfendiyarov Kazakh National Medical University, 94 Tole-bi St., Almaty 050000, Kazakhstan; balabekova.m@kaznmu.kz (M.K.B.); limariya2000@gmail.com (M.L.); alimova.milana1999@gmail.com (M.A.A.); muslim.m@kaznmu.kz (M.B.M.); 2 Laboratory of Synthetic and Natural Medicinal Compounds Chemistry, A.B. Bekturov Institute of Chemical Sciences, 106, Ualikhanov str., Almaty 050010, Kazakhstan; symba_t@mail.ru (S.Z.); kh.tassibekov@ihn.kz (K.S.T.); ramazanova.akerke@bk.ru (A.A.R.); 3 Laboratory of Engineering Profile NMR Spectroscopy, Sh. Ualikhanov Kokshetau State University, 76 Abai St., Kokshetau 020000, Kazakhstan; tseilkhanov@mail.ru

**Keywords:** acute pneumonia, T lymphocytes, interleukin-4, transforming growth factor-β (TGF-β), experiment, Complex of 1-(2-Ethoxypropyl)-4-(pent-1-yn-1-yl)piperidin-4-yl Propionate with β-Cyclodextrin (β-CD)

## Abstract

**Background/Objectives:** Acute pneumonia remains one of the leading causes of mortality worldwide. The pathogenesis of the disease is determined by the nature of the host immune response. The balance between effector and regulatory T cells (Treg) is critical, as it determines the severity of inflammation and the regenerative capacity of lung tissue. The development of new approaches to modulate the immune response using promising synthetic compounds opens up the possibility of targeted cytokine balance restoration of cytokine balance and Tregs functions This study investigated the effects of the newly synthesized complex of 1-(2-Ethoxypropyl)-4-(pent-1-yn-1-yl)piperidin-4-yl Propionate with β-Cyclodextrin (MXF-22), on the populations of CD4^+^, CD4^+^CD25^+^ and CD4^+^FoxP3^+^ T cells in an oleic acid-induced acute lung injury rat model. **Methods:** Quantitative analysis of CD4^+^, CD4^+^CD25^+^, and CD4^+^FoxP3^+^ T cell subsets and serum IL-4 and TGF-β levels were determined by flow cytometry and ELISA assays, respectively. **Results:** The study revealed a significant decrease in the number of CD4^+^ T cells and their regulatory subsets (CD4^+^CD25^+^, CD4^+^FoxP3^+^) during acute pneumonia. Oral administration of MXF-22 contributed to a pronounced recovery of these parameters, accompanied by increased levels of IL-4 and TGF-β, which indicated the activation of anti-inflammatory and reparative processes. **Conclusions:** MXF-22 showed a pronounced immunomodulatory effect contributing to the restoration of the function of CD4^+^ FoxP3^+^ T regs in acute pneumonia rat model.

## 1. Introduction

Acute lung injury is a common severe inflammatory disorder of the lungs characterized by acute respiratory distress and hypoxemia due to impaired oxygenation. One of its early manifestations is diffuse alveolar lesion [1,2]. CD4^+^FoxP3^+^ regulatory T cells (Treg) play a key role in maintaining tissue homeostasis and controlling immune responses. Their functional activity and clinical significance have been investigated in various pathological conditions, from autoimmune and inflammatory diseases to oncological diseases [3,4]. These cells are characterized by the expression of the transcription factor FoxP3, which suppresses an excessive immune response through the secretion of anti-inflammatory cytokines such as interleukin-4 (IL-4) and transforming growth factor β (TGF-β), as well as through direct intercellular interactions [5,6,7]. Tregs, identified by intracellular expression of FoxP3, migrate to the lungs during the immune response in a mouse model of Pneumocystis pneumonia, while the loss of this cell population is associated with increased disease severity [8,9,10].

CD4^+^FoxP3^+^ Tregs play a central role in maintaining immunological tolerance and suppressing excessive inflammation. In this context, cytokines IL-4 and TGF-β act as key mediators with which Tregs either interact directly or rely on their functions. Regulatory T cells differentiate from Th cells in the presence of TGF-β [11]. Although TGF-β signaling is not required for maintenance of Treg homeostasis, suppression of T cells proliferation, or inhibition of type 1 T helper cells (Th1) differentiation by Treg cells is essential for effective suppression of Th17 responses and the regulation of tissue-specific immune reactions [12]. TGF-β is a key cytokine that regulates multiple aspects of the T cell lifecycle, including their development, activation, proliferation, differentiation, and apoptosis. In CD4^+^ T cells, TGF-β maintains a quiescent state and limits the excessive activation of naive cells. At the same time, it suppresses the differentiation and activity of Th1 and Th2, while promoting the formation of Th17 and Th9 cells. Importantly, TGF-β is indispensable for the induction of FoxP3 expression in naive CD4^+^ T cells and in the development of Tregs. Additionally, TGF-β signaling is essential in restriction of effector T cell functional activity to prevent uncontrolled immune response [12,13,14,15,16].

The literature contains conflicting data regarding the role of IL-4. Several studies report that IL-4 suppresses TGF-β–induced FoxP3 expression and inhibits the generation of new Treg cells. Together, these cytokines induce Foxp3^−^ IL-9^+^ IL-10^+^ T cells, which lack suppressive function but are capable of enhancing inflammation and activating effector T cells [17]. In addition, it has been shown that IL-4 not only inhibits the development of induced Tregs but also reduces the level of FoxP3 mRNA and decreases the number of FoxP3^+^ cells in the population of these Tregs [18,19]. At the same time, a number of studies describe the role of IL-4 in the regulation of Treg cells and immunosuppression. A physiologically significant concentration of IL-4 may contribute to Treg-mediated immunosuppression, possibly by enhancing granzyme expression [20,21].

The development of new approaches for immune response modulation using synthesized compounds enables targeted restoration of cytokine balance and enhancement of regulatory T cells function, which opens prospects for prevention of hyperinflammatory complications and acceleration of reparative processes in inflammatory diseases. Therefore, in this study, a model of acute pneumonia caused by intravenous oleic acid was used to study the role of Tregs in modulating the host response, as well as to perform pathogenetic correction with the newly synthesized compound MXF-22.

The selection of MXF-22 was justified by our long-term research dedicated to the biological properties of piperidine- and piperazine-derived compounds, such as MXF-2—1-(2-ethoxyethyl)-4-(dimethoxyphosphoryl)-4-hydroxypiperidine (an oxyphosphonate) [22], MXF-18—1-(2-ethoxyethyl)-4-(pent-1-yn-1-yl)piperidin-4-yl propionate (EPPP), and its 1:1 complex with β-CD (EPPPβCD) [23], MXF-19—complex of dimethyl[(4-benzhydrylpiperazin-1-yl)(2-fluorophenyl)methyl]phosphonate with β-CD ((o-Fph)PPhβCD) [24,25], and MXF-20—1-[1-(2,5-dimethoxyphenyl)-4-(naphthalene-1-yloxy)but-2-ynyl]-4-methylpiperazine, in complex with β-CD (the complex) [26,27].

The biological effects of these compounds were studied using models of acute pneumonia in some experiments [25] and aseptic inflammation induced against a background of prior intoxication with vanadium and chromium salts [22,23], or cadmium and lead salts [24,26,27] in others. A comprehensive assessment of immune-mediated regulatory disturbances in aseptic inflammation and acute pneumonia was performed using hematological, immunological, and microscopic analyses of the inflammatory focus [28]. Key impairments in immune regulation were evaluated by analyzing splenic subcellular populations with the phenotypes His48^+^CD11b/c^+^, His48^High^CD11b/c^+^, His48^low^CD11b/c^+^, CD3^+^CD4^+^, CD3^+^CD4^+^IFNγ^+^, CD3^+^CD4^+^IL-4^+^, CD8^+^, CD4^+^CD25^+^, CD4^+^CD25^+^FoxP3^+^, CD4^+^FoxP3^+^CTLA^+^, B220^+^RT1^+^, RT1^+^ in CD45^+^CD45R(B220)^+^, as well as through the quantitative analysis of pro- and anti-inflammatory cytokines (IL-1β, IL-6, IL-10, and TGF-β) [22,23,24,25,26,27,28].

As previously described, MXF-2 (the oxyphosphonate) exhibited pronounced regenerative and immunomodulatory activity, including stimulation of proliferative activity in CD4^+^ and CD8^+^ lymphocytes [22]. Similar effects were identified for MXF-18 (EPPP) and its β-CD complex, which enhanced proliferation and phagocytosis of lymphomonocytic cells and modulated Treg activity at early stages of inflammation [23]. The MXF-19 complex ((o-Fph)PPhβCD) demonstrated immunomodulatory effects both in metal-induced aseptic inflammation and in the acute pneumonia model, leading to normalization of CD4^+^ and CD4^+^CD25^+^ populations and a reduction in CD4^+^CD25^+^FoxP3^+^ T cells [24,25]. Subsequent evaluation of MXF-20 further revealed its potential to modulate myeloid-derived suppressor cell activity even under combined cadmium and lead toxicity. Moreover, MXF-20 improved wound healing through immunomodulation, stimulation of granulation tissue formation, and enhancement of vascular regeneration [26,27].

The identification of cumulative mechanisms underlying the impaired regulatory influence of these key immune pathways on the inflammatory process, along with pathogenetic correction, demonstrated that these compounds significantly accelerated inflammation resolution, confirming their biological effectiveness at early research stages. Considering that the structurally related piperidine derivative EPPPβCD had previously exhibited a strong ability to accelerate the resolution of inflammation induced by chromium and vanadium salts—largely through its substantial effects on regulatory T-lymphocyte activity—we found it necessary to continue investigating the next compound in the series, MXF-22. This allowed us to determine whether it possesses comparable or superior immunomodulatory potential in a model of acute pneumonia not associated with metal-induced immunosuppression, and to evaluate its impact on Treg-mediated mechanisms of anti-inflammatory control.

We have clearly demonstrated that MXF-22, along with the reference drug Polyoxidonium, contributes to the expansion of Treg and the resolution of lung inflammation.

## 2. Materials and Methods

### 2.1. Chemical Experimental Part

#### The Synthesis and Structure Studies for the Chemical Compounds

Reagents used in synthesis were purchased from Merck (Boston, MA, USA) and included Propionic anhydride, ≥99% (CAS No. 123-62-6); Propionyl chloride, 98% (Cas No. 79-03-8). The 1-(2-ethoxypropyl)-4-(pent-1-yn-1-yl)piperidin-4-ol was synthesized, purified, and identified. The progression of reactions and the purity of the synthesized products were assessed using thin-layer chromatography (TLC) on alumina (Al_2_O_3_) plates. The spots of the substances were visualized with iodine vapor. For TLC, a benzene–dioxane mixture (5:1) was used as the eluent. Elemental analysis was conducted using a Rapid Micro N Cube elemental analyzer to confirm the composition of the products. FT-IR spectral analysis was performed using a NICOLET 5700 FT-IR spectrometer (Thermo Fisher Scientific, Madison, WI, USA) in the 400–4000 cm^−1^ range. Spectra were acquired and processed using the OMNIC software package v.7.1 (Thermo Electron Corporation, Madison, WI, USA). Liquid samples were applied as thin films on KBr plates, whereas solid samples were analyzed as KBr pellets. Additionally, the ^1^H and ^13^C NMR spectra of the samples were recorded using a JNM-ECA 400 (JEOL, Tokyo, Japan) spectrometer, operating at frequencies of 399.78 MHz for ^1^H and 100.53 MHz for ^13^C, in deuterated chloroform (CDCl_3_) and dimethyl sulfoxide (DMSO-d_6_). Chemical shifts are measured relative to the signals of residual protons or carbon atoms of the deuterated solvent as standard (the NMR spectra and their detailed interpretation are provided in the Supplementary Materials).

The Synthesis of *1-(3-Ethoxypropyl)-4-(pent-1-yn-1-yl)piperidin-4-ol* (**EPP-ol**). A mixture of KOH (15.15 g, 0.2705 mol), a trace amount of hydroquinone, and pent-1-yne (18.39 g, 0.2705 mol) in dry benzene was stirred, and a benzene solution of 1-(3-ethoxypropyl)-4-oxopiperidine (10.0 g, 0.0541 mol) was added dropwise. After completion of the reaction (TLC control), 22.7 mL of water was added, and the mixture was extracted with diethyl ether. The combined organic layers were washed with 10% HCl, dried over anhydrous MgSO_4_, filtered, and concentrated under reduced pressure.

The crude product was purified by standard work-up to afford **EPP-ol** as a light-yellow oil (9.18 g, 67.2%), Rf = 0.27 (benzene/dioxane 5:1).

The elemental composition of **EPP-ol**, C_15_H_27_NO_2_, was calculated as follows: Carbon (C) 71.10%, Hydrogen (H) 10.74%, Nitrogen (N) 5.53%. The analytical results found were as follows: Carbon (C) 71.22%, Hydrogen (H) 10.62%, Nitrogen (N) 5.67%.

IR (KBr, ν, cm^−1^): see Figure S1.

^1^H NMR and ^13^C NMR (DMSO-d_6_, δ, ppm): Figures S5–S10.

The Synthesis of *1-(3-Ethoxypropyl)-4-(pent-1-yn-1-yl)piperidin-4-yl Propionate* (**EPP-pr**). 1-(3-Ethoxypropyl)-4-(pent-1-yn-1-yl)-4-hydroxypiperidine (**EPP-ol**) (3.0 g, 0.0119 mol) was treated with propionic anhydride (15.42 g, 0.119 mol) and propionyl chloride (10.97 g, 0.119 mol). The reaction mixture was kept at room temperature for 3 days, after which excess reagents were removed under reduced pressure. The residue was treated with water and neutralized with potassium carbonate. The organic phase was extracted with benzene, dried over anhydrous MgSO_4_, filtered, and concentrated.

The product **EPP-pr** was obtained as a light-yellow oil (4.4 g, 88.5%), Rf = 0.94 (benzene/dioxane 5:1).

The elemental composition of **EPP-pr**, C_18_H_31_NO_3_, was calculated as follows: Carbon (C) 69.87%, Hydrogen (H) 10.10%, Nitrogen (N) 4.53%. The analytical results found were as follows: Carbon (C) 69.87%, Hydrogen (H) 10.94%, Nitrogen (N) 4.75%.

IR (KBr, ν, cm^−1^): see Figure S2.

^1^H NMR and ^13^C NMR (DMSO-d_6_, δ, ppm): see Table S1 and Figures S11–S16.

*The Complex of 1-(2-Ethoxypropyl)-4-(pent-1-yn-1-yl)piperidin-4-yl Propionate with β-CD* (**MXF-22**) *Synthesis*. A solution of **EPP-pr** (0.6 g, 0.0019 mol) in ethanol (30 mL) was mixed with an aqueous solution of β-CD (2.2 g, 0.0019 mol in 50 mL distilled water). The mixture was placed in a drying oven at 50–55 °C until complete removal of ethanol and water (6 h). The resulting β-CD inclusion complex **(MXF-22)** was obtained as a white powder (2.60 g, 93%), decomposing above 240 °C.

The elemental composition of **MXF-22**, C_59_H_99_O_38_N, was calculated as follows: Carbon (C) 49.54%, Hydrogen (H) 6.98%, Nitrogen (N) 0.98%. The analytical results found were as follows: Carbon (C) 49.50%, Hydrogen (H) 6.98%, Nitrogen (N) 1.05%.

IR (KBr, ν, cm^−1^): see Figure S3.

^1^H NMR and ^13^C NMR (DMSO-d_6_, δ, ppm): see Table S1 and Figures S17–S25.

### 2.2. Animals

The subjects were outbred male rats (*n* = 60) aged 8–12 weeks and weighing 160–200 g, housed in standard vivarium conditions (room temperature 22.5–23.0 °C, relative humidity 50–70%, 12 h day/night cycle). Animals were provided with standard animal diet and water. All experimental procedures were conducted in accordance with the Rules for Conducting Biomedical Experiments, Preclinical (Non-clinical), and Clinical Studies, and Requirements for Preclinical and Clinical Facilities approved by Order No. 142 of the Minister of Health of the Republic of Kazakhstan, dated 2 April 2018 (registered with the Ministry of Justice of the Republic of Kazakhstan on 17 April 2018, No. 16768) [29] and were approved by the bioethics committee of the Kazakh National Medical University named after S. D. Asfendiyarov (protocol No. 25 (161) dated 28 March 2025).

### 2.3. Compounds

The study utilized two compounds for acute pneumonia treatment in experimental models. The investigational compound was a complex of 1-(2-Ethoxypropyl)-4-(pent-1-yn-1-yl)piperidin-4-yl Propionate with β-CD (MXF-22), supplied by the Laboratory of Synthetic and Natural Medicinal Compounds Chemistry, A.B. Bekturov Institute of Chemical Sciences (Almaty, Kazakhstan). MXF-22 was given in a volume of 325 mg/kg of body weight. Polyoxidonium (azoximer bromide), at dose 0.32 mg/kg (as according to the manufacturer’s instructions), served as the comparator drug [30].

The choice of Polyoxidonium as the comparator drug for MXF-22 was based on our previously established findings regarding its immunomodulatory properties. Our earlier studies showed that ammonium metavanadate and potassium dichromate increase the proportion of B220^+^RT1^+^ splenocytes. At later stages of inflammation, the proportion of Treg cells and their expression of suppressor markers increased. Exposure to vanadium and chromium salts, as well as the combined impact of metals and aseptic inflammation, led to a chronic elevation in Treg cells expressing FoxP3^+^ and CTLA-4^+^. Meanwhile, our results demonstrated that Polyoxidonium exerts a modulatory effect on B-lymphocytes and inhibits Treg-cell activity during aseptic inflammation induced by ammonium metavanadate and potassium dichromate [31].

In addition, published evidence indicates that azoximer bromide stimulates T-cell proliferation and enhances the expression of costimulatory molecules on innate immune cells, accompanied by the activation of signaling cascades, including NF-κB, and increased cytokine production. These changes indirectly promote B-cell activation and the development of an adaptive immune response. After entering the endosomal compartment, the compound elevates the level of micromolecular hydrogen peroxide, activating signaling pathways and transcription factors such as NF-κB, which mediate its antioxidant effects. Together, these mechanisms underlie the complex immunomodulatory activity of Polyoxidonium, enhancing the functional activity of both innate and adaptive immune cells [32].

Taken together, these considerations justify the use of Polyoxidonium as a mechanistically appropriate and experimentally validated comparator for assessing the immunomodulatory activity of MXF-22 in our model.

### 2.4. Experimental Design

**Figure 1:**
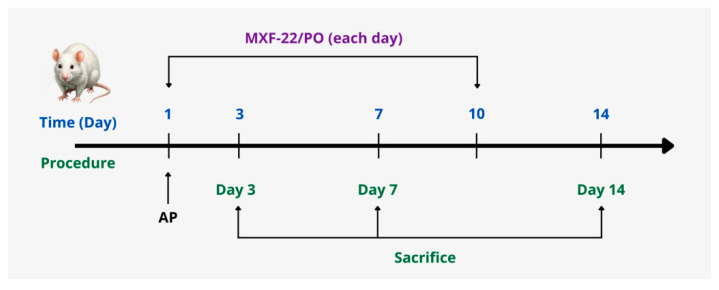
Experimental design.

Rats were randomly assigned into 4 groups. Each group contained 18 animals, with the exception of the control group, which included 6 animals. Group C (control): intact animals without any intervention or treatment. Group AP (acute pneumonia): animals with oleic acid-induced acute pneumonia that did not receive any treatment. Group AP/PO: animals with oleic acid-induced acute pneumonia that received treatment with Polyoxidonium. Group AP/MXF-22: animals with oleic acid-induced acute pneumonia that received MXF-22. The treatment with either Polyoxidonium or MXF-22 was given for 10 consecutive days (Figure 1).

To study acute lung injury, the oleic acid-induced model was used in this research. The model reproduces the key signs of acute pneumonia—neutrophilic inflammation, increased vascular permeability, alveolar edema, and damage to the epithelial and endothelial barrier [33]. In the acute experiment, the model provides a complete picture of acute pneumonia development, excluding the incubation period, and provides a short period of injury formation. Although there are several limitations of the model associated with the absence of an infectious component, the incomplete reproduction of specific adaptive reactions and long-term interaction of the immune system with the pathogen, the model is safe, standardized, and allows effective study of the anti-inflammatory and regenerative effects of the compound.

Animals in groups AP, AP/PO, and AP/MXF-22 were injected intravenously via a tail vein with 0.25 mL of suspension of oleic acid (OA) in bovine serum albumin (BSA) to induce acute pneumonia [32]. The solution was prepared by the vigorous mixing of OA in a 0.1 mg/mL (*w*/*v*) solution of BSA in sterile physiological saline at a volumetric ratio of 1:5. Group C was given an equal volume of physiological saline.

All manipulations were conducted under the general anesthesia with Zoletil and Xylazine at doses of 20–40 mg/kg and 5–10 mg/kg, respectively, administered intramuscularly. Atropine was given intramuscularly at 0.5–1 mg/kg as premedication beforehand [33].

After the procedure, the animals were placed back in their cages and monitored until they had completely recovered from anesthesia.

The animals were orally administered a suspension of MXF-22 and Polyoxidonium. The MXF-22 suspension was prepared by diluting 325 mg of the compound in 10 mL of saline solution (NaCl). Based on the calculation that 1 mL of the suspension contains 32.5 mg of MXF-22, a 200 g rat was given 2 mL of the suspension. A suspension of Polyoxidonium was prepared by diluting 1.28 mg of the compound in 20 mL of saline solution (NaCl). Based on the calculation that 0.5 mL of the suspension contains 0.032 mg of Polyoxidonium, a 200 g rat was given 1 mL of the suspension.

Assessments were performed on days 3, 7, and 14 after the initiation of pneumonia. Animals were euthanized under the general anesthesia with Zoletil–Xylazine, with prior atropine premedication [34]. At each point of time, 6 control animals (one-time collection) and 6 rats per group were sacrificed. The blood samples and spleens were collected for immunological analysis.

Quantitative analysis of CD4^+^, CD4^+^CD25^+^, and CD4^+^FoxP3^+^ T cell subpopulations and serum IL-4 and TGF-β levels were evaluated by flow cytometry and ELISA, respectively, in the Life Science laboratory B.A. Atchabarov Institute of Fundamental and Applied Medicine at Asfendiyarov Kazakh National Medical University.

### 2.5. Flow Cytometry

(1) The markers were determined using flow immunocytofluorometry. For this purpose, the cell suspension was treated with monoclonal antibodies to surface markers, according to the manufacturers’ protocols. The sample was preparation and staining of splenocytes for flow cytometry. The spleen (approximately 0.5 × 2 cm in size) was placed in 0.5 mL of cold physiological saline. After that the homogenized tissue was mixed with 4.5 mL of cold solution, filtered through a nylon filter (70 μm), and centrifuged at 400 G for 5 min, then the supernatant was carefully removed with a pipette. Lysis of erythrocytes was performed by 10 min incubation of the cells in 2 mL of High-Yield Lyse reagent, with preliminary mixing of the solution, splenocytes were in solution, the liquid was carefully taken into micro-samples, without affecting pieces of tissue, large fragments of suspension, and other elements of the solution. Then, cells were washed two times in physiological saline and resuspended in 100 μL of cold solution. Centrifugation was carried out at 300 RCF for 5 min with the addition of 1.5 mL of 0.9% saline solution. Surface staining was performed using monoclonal antibodies against the respective markers according to the manufacturer’s protocol. A mixture of antibodies was added to the prepared cell suspension: 60 μL of anti-CD4 and 30 μL of anti-CD25 antibodies diluted in 810 μL of 0.9% saline. The sample was thoroughly mixed and incubated for 30 min at +4 °C in the refrigerator. The fixation/permeabilization solution was prepared by mixing 15 mL of FoxP3 fixation/permeabilization concentrate with 45 mL of FoxP3 fixation/permeabilization diluent. Cells were incubated for 30 min at room temperature in the dark. To prepare the permeabilization buffer, 5.1 mL of 10× Permeabilization Buffer was diluted in 45.9 mL of distilled water. Cells were washed three times followed by centrifugation at 500 RCF for 5 min, after which the supernatant was removed. A mixture of antibodies for intracellular staining was added to the pellet and mixed thoroughly. The intracellular antibody mixture was prepared by adding 90 μL of anti-FoxP3 to 810 μL of saline. Then, 15 μL of this antibody solution was added to each sample. Samples were incubated at room temperature for 30 min. Next, 1.5 mL of 1× Permeabilization Buffer was added, followed by centrifugation at 400 RCF for 5 min, and the supernatant was removed. Finally, 500 μL of 0.9% saline was added, and the pellet was resuspended by vortexing or pipetting. The samples were analyzed using a flow cytometer. Following staining, cells were washed, fixed, and resuspended in 500 µL of 0.9% saline. Non-specific fluorescence was assessed using fluorescence-minus-one (FMO) controls.

Prepared samples were analyzed on an Attune™ NxT flow cytometer (Thermo Fisher Scientific, Waltham, MA, USA), evaluating the percentage of labeled cells, including major T-lymphocyte populations (CD4^+^, CD4^+^CD25^+^, CD4^+^FoxP3^+^) (Table 1). Flow cytometry data were processed with Attune™ NxT Cytometrix Software, v6.0.1 (Thermo Fisher Scientific, Waltham, MA, USA).

(2) T-lymphocyte Gating Protocol.

FSC vs. SSC: Lymphocyte gating was performed based on their size and granularity.
CD4^+^ T-lymphocytes:
CD4^+^ cells were identified using antibodies against CD4.
Regulatory T-cells (Treg):
CD4^+^CD25^+^: CD25 expression was evaluated within the CD4^+^ gate.
CD4^+^FoxP3^+^: FoxP3 intracellular staining was conducted within the CD4^+^ gate.

### 2.6. IL-4 and TGF-β ELISA Assays

The blood samples were obtained via cardiac puncture, followed by serum isolation through centrifugation. Serum IL-4 and TGF-β levels were measured using the ELISA kits (Invitrogen, Thermo Fisher Scientific, Waltham, MA, USA) (Table 2).

IL-4

Microplate wells are coated with anti-IL-4 antibodies. Rat IL-4 presents in the sample or standard binds to these antibodies. A biotin-conjugated anti-IL-4 antibody is then added, binding to the captured IL-4. Following incubation, unbound biotin-conjugated antibodies are removed by washing. Streptavidin-HRP is added, attaching to the bound biotin-conjugated anti-IL-4 antibodies. After incubation, excess Streptavidin-HRP is removed by washing. A substrate solution is added, which reacts with HRP, forming a colored product whose intensity is proportional to the amount of IL-4 in the sample. The reaction is stopped by the addition of acid, and absorbance is measured at 450 nm. A standard curve is generated using seven serial dilutions of rat IL-4, allowing determination of IL-4 concentration in the samples.

TGF-β

Microplate wells are coated with anti-TGF-β1 antibodies. Rat TGF-β1 present in the sample or standard binds to these antibodies. After incubation, unbound components are removed by washing. A biotin-conjugated anti-TGF-β1 antibody is then added, binding to the captured TGF-β1. Following incubation, unbound biotin-conjugated antibodies are washed away. Streptavidin-HRP is added, binding to the biotin-conjugated anti-TGF-β1 antibodies. Excess Streptavidin-HRP is removed by washing. A substrate solution is added, producing a colored reaction product proportional to the TGF-β1 concentration in the sample or standard. The reaction is stopped by acid addition, and absorbance is measured at 450 nm. A standard curve is generated using seven serial dilutions of rat TGF-β1 to determine TGF-β1 concentration in the samples.

### 2.7. Statistical Analysis

All experiments were performed in at least 6-fold repetition. Statistical analyses were performed using a two-way ANOVA with a post hoc Tukey test. If *p* value was ≤0.05, the difference was considered statistically significant.

The Graphpad Prism v.10 program was used to perform statistical analysis, visualize and obtain graphical images. Graphs and figures contain information in the form of arithmetic averages M/(standard deviation, SD).

## 3. Results

Two main reasons motivated the present Research. The first lies in the high pharmacological potential of the N-alkoxyalkylethynylpiperidine fragment, as previously demonstrated by Satbayeva E.M. et al. [35]. The second and primary reason is our recent finding that the β-CD complex of 1-(2-ethoxyethyl)-4-(pent-1-yn-1-yl)piperidin-4-yl propionate markedly accelerates the resolution of inflammation induced by chromium and vanadium salts intoxication [23].

**Scheme 1:**
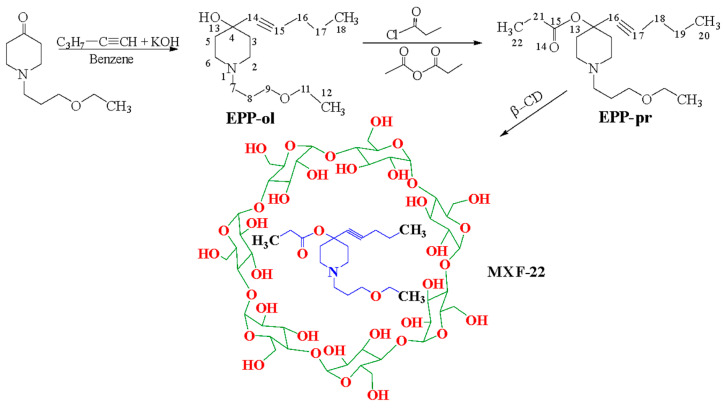
Synthesis of 1-(2-ethoxypropyl)-4-(pent-1-yn-1-yl)piperidin-4-yl propionate (EPP-pr) and its complex (MXF-22).

A series of synthetic transformations was carried out, beginning with the ethynylation of 1-(2-ethoxypropyl)-4-oxopiperidine with pent-1-yne, which afforded the corresponding 4-(pent-1-yn-1-yl)piperidin-4-ol (**EPP-ol**) in a 67.2% yield. The resulting alcohol was then treated with a mixture of propionyl chloride and propionic anhydride, giving the target propionate ester (**EPP-pr**) in an 88.5% yield (Scheme 1).

The IR spectra of the piperidinol **EPP-ol** (Figure S1), and its propionate ester **EPP-pr** (Figure S2), recorded as KBr films, displayed characteristic absorption bands at 2236.8 and 2246.6 cm^−1^, corresponding to the C≡C stretching vibration. **EPP-ol** exhibited an O–H stretching band at 3405.4 cm^−1^, whereas **EPP-pr** showed a strong carbonyl absorption at 1746.1 cm^−1^, consistent with ester formation.

Detailed ^1^H and ^13^C NMR spectra of **EPP-ol** (Figures S5–S10) and **EPP-pr** (Figures S11–S16) are provided in the Supplementary Materials. Additionally, the structures of the piperidinol and the corresponding propionate ester were confirmed using two-dimensional NMR techniques, including COSY (^1^H–^1^H) (Figures S8 and S14), HMQC (^1^H–^13^C) (Figures S9 and S15), and HMBC (^1^H–^13^C) (Figures S10 and S16), which reveal the key homo- and heteronuclear spin–spin correlations, as illustrated in Figures S7 and S13.

The compound 1-(2-ethoxypropyl)-4-(pent-1-yn-1-yl)piperidin-4-yl propionate is an oily substance, and its incorporation into β-CD allows its transformation into a stable solid powder. The inclusion process improves the compound’s stability in aqueous media, decreases oxidative degradation upon exposure to air, and reduces dehydration and volatilization. Formation of the β-CD inclusion complex is expected to increase the bioavailability of the propionate derivative while lowering its toxicity. These advantages indicate that β-CD complexation is a practical approach to enhancing the pharmacological characteristics of piperidine-based molecules intended for biomedical applications.

The inclusion complex was prepared by mixing an ethanolic solution of the propionate with an aqueous solution of β-CD in a 1:1 molar ratio. The mixture was dried in an oven at 50–55 °C until complete solvent removal, yielding the inclusion complex as a white powder with a 93.0% yield (Scheme 1).

To confirm inclusion complex formation, both FT-IR (Figures S3 and S4) and NMR spectroscopy (Figures S17–S24) were employed by comparing the spectral data of the starting materials (**EPP-pr** and β-CD) with those of the final product (**MXF-22**). Comparative FT-IR spectra of **EPP-pr** and β-CD are shown in Figures S3 and S4 demonstrating that the spectrum of **MXF-22** contains absorption bands characteristic of both components. In contrast, the ^1^H NMR data (Figure S17 and Table S1) provided more definitive evidence for the formation of the target inclusion complex. Based on the infrared spectrum of **MXF-22**, it can be inferred that the propionate molecule enters the β-CD cavity with its N-ethoxypropylpiperidine moiety.

Additional evidence for inclusion complex formation was obtained from thin-layer chromatography on aluminum oxide (eluent: benzene/dioxane = 5:1). After 6 h of incubation at 50–55 °C, the spot corresponding to **EPP-pr** (Rf = 0.94) disappeared, further supporting the formation of the **MXF-22** complex.

**Figure 2:**
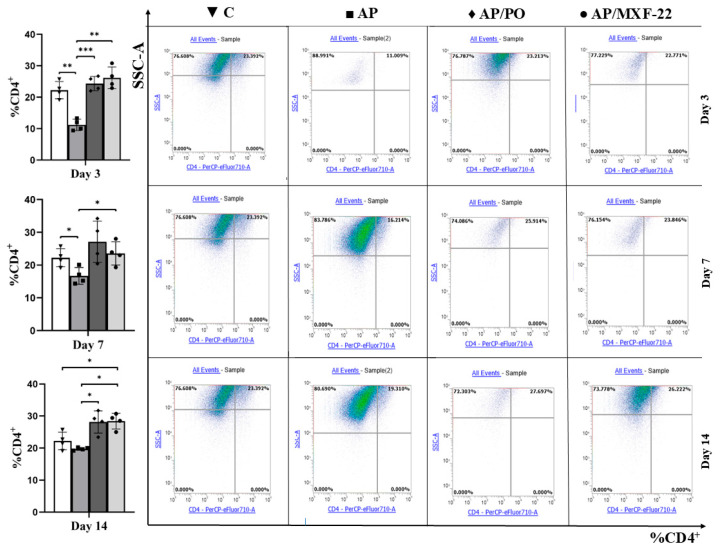
The effect of Polyoxidonium and the new synthetic compound MXF-22 on CD4^+^ lymphocyte count in acute pneumonia. After splenocyte isolation, the cells were stained with antibodies conjugated with fluorochromes, followed by analysis by flow cytometry. The figure shows dot diagrams (days 3, 7, 14) reflecting the distribution of CD4^+^; bar charts show the average values of the proportion of CD4^+^ cells (%) in the rat spleen on the 3rd, 7th, and 14th days of the experiment. Groups: **C**—control; **AP**—acute pneumonia without treatment; **AP/PO**—Polyoxidonium treatment; **AP/MXF-22**—MXF-22 treatment. Statistics were performed using two-way ANOVA, with multiple comparisons corrected with Tukey’s method. All data is presented as an average value ± standard deviation (*n* = 6). * *p* < 0.05; ** *p* < 0.01; *** *p* < 0.001.

The experimental animals were divided into four groups: C (control)—intact animals without any intervention or treatment; AP (acute pneumonia)—animals with induced acute pneumonia without treatment; AP/PO—animals with acute pneumonia treated with Polyoxidonium; AP/MXF-22—animals with acute pneumonia treated with the new synthetic compound MXF-22. In the spleen of experimental animals, we investigated differences in the percentage of CD4+ T cells (*n* = 6) using flow cytometry. The presence of the CD4 surface marker is a universal sign of mature T cells involved in the regulation of the immune response. Reduced CD4^+^ lymphocyte counts generally reflects inhibition of the T-cell–mediated immune response, while elevated levels suggest activation of cellular immunity or recovery of immune function after an inflammatory process. A new synthesized compound MXF-22 was used on the acute pneumonia model, comparing it with the well-known immunomodulatory drug Polyoxidonium [30,31]. The results of flow cytometry performed during certain periods of the experiment (3, 7, 14 days) showed a noticeable improvement in the dynamics of CD4+ T cells (Figure 2).

In the AP group, a statistically significant decrease in the proportion of CD4^+^ lymphocytes was observed compared with the control group at all follow-up time points. After 3 days, the content of CD4^+^ cells decreased by more than two-fold (*p* = 0.004), indicating pronounced inhibition of the T-cell component of the immune response in the acute period of inflammation. By day 7, the CD4^+^ cell level remained reduced, amounting to approximately 75% of the control level (16.7% vs. 22.2%; *p* = 0.045), and by day 14 a notable trend toward the recovery of CD4^+^ cell levels were noted—the value reached about 89% of the control and no statistical difference was found between these groups. (19.8% vs. 22.2%; *p* = 0.4208). Polyoxidonium correction was accompanied by a gradual increase in CD4^+^ cell levels compared with the AP group: a 2.2-fold increase on day 3 (24.3% vs. 11.2%; *p* = 0.0006) and by day 14 it exceeded the control value by approximately 27% *p* = 0.048), indicating activation of immune restoration processes. MXF-22 induced a similar, and in some cases a more pronounced, response. In comparison with the AP group, in AP/MXF-22, the proportion of CD4^+^ cells was approximately 15% higher on day 3 (26.2% vs. 11.2%, *p* = 0.003), 6.87% higher on day 7 (23.6% vs. 16.7%, *p* = 0.047), and 8.7% higher on day 14 (28.4% vs. 19.9%, *p* = 0.015), indicating stimulation of immune correction processes. Moreover, the levels of CD4^+^ cells were significantly higher on day 14 in AP/MXF-22 group compared with the control group (28.4% vs. 22.2%, *p* = 0.05).

On days 3 and 7, CD4^+^ levels in the AP/MXF-22 group exceeded those in the AP group by 2.3-fold (*p* = 0.003) and 1.4-fold (*p* = 0.047), respectively, approaching the control values. By day 14, the proportion of CD4^+^ cells with MXF-22 treatment was comparable to that observed with Polyoxidonium, indicating the persistence of the activating effect and a potential normalization of the T-cell balance under the influence of MXF-22.

**Figure 3:**
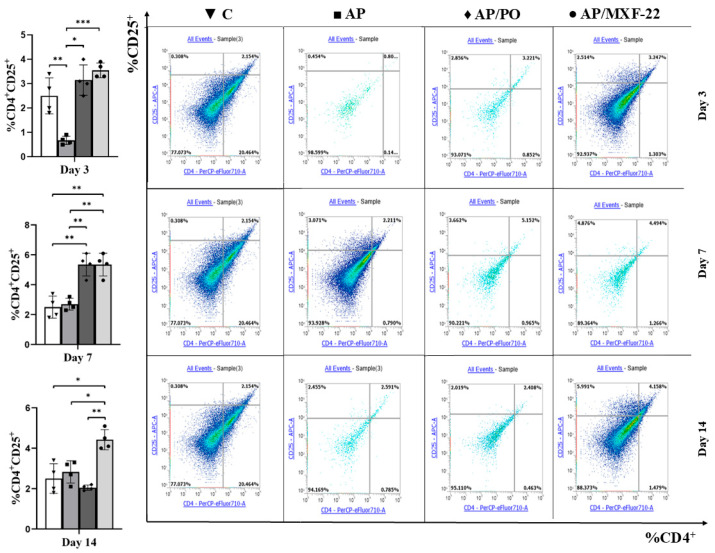
The effect of Polyoxidonium and the new synthetic compound MXF-22 on CD4^+^CD25^+^ lymphocyte count in acute pneumonia. After splenocyte isolation, the cells were stained with antibodies conjugated with fluorochromes, followed by analysis by flow cytometry. The figure shows dot diagrams (days 3, 7, 14) reflecting the distribution of CD4^+^CD25^+^ cells; bar charts show the average values of the proportion of CD4^+^CD25^+^ lymphocytes (%) in the rat spleen on the 3rd, 7th, and 14th days of the experiment. Groups: **C**—control; **AP**—acute pneumonia without treatment; **AP/PO**—Polyoxidonium treatment; **AP/MXF-22**—MXF-22 treatment. Statistics were performed using two-way ANOVA, with multiple comparisons corrected with Tukey’s method. All data is presented as an average value ± standard deviation (*n* = 6). * *p* < 0.05; ** *p* < 0.01; *** *p* < 0.0001.

To provide a more comprehensive assessment of T-cell immunity in acute pneumonia, we additionally evaluated the population of CD4^+^CD25^+^ lymphocytes, which reflects regulatory T-cell (Treg) activity and their role in limiting the inflammatory response. The results are presented in Figure 3.

In rats with acute pneumonia, there was a significant decrease in the content of CD4^+^CD25^+^ lymphocytes relative to the control group. When analyzing the relative content of CD4^+^CD25^+^ lymphocytes, it was found that after 3 days in animals with acute pneumonia, there was a statistically significant 3.6-fold decrease in the proportion of regulatory T cells compared with the control (0.7% vs. 2.5%; *p* = 0.041), suggesting significant down-regulation of the Treg axis of the immune response in the acute phase of inflammation. After 7 and 14 days, a trend toward recovery of this parameter was observed (2.7% and 2.8%, respectively), although full restoration was not achieved. Polyoxidonium significantly increased the content of CD4^+^CD25^+^ lymphocytes relative to the AP group. After 3 days, their fraction increased more than 4-fold (3.2% vs. 0.7%; *p* = 0.0091), and after 7 days doubled (5.4% vs. 2.7%; *p* = 0.008), indicating activation of the regulatory arm of T-cell immunity. Moreover, on day 7 CD4^+^CD25^+^ cell levels in the AP/PO group exceeded the values of the control group by 2.1-fold (5.4% vs. 2.5%, *p* = 0.007). By day 14, CD4^+^CD25^+^ cell levels returned to control values which may reflect completion of the active phase of immune recovery. The use of MXF-22 led to a more pronounced and sustained increase in the proportion of CD4^+^CD25^+^ lymphocytes. After 3 days, their content increased 5-fold compared with the AP group (3.6% vs. 0.7%; *p* ≤ 0.0001), by day 7 it remained at a high level—2 times higher (5.4% vs. 2.7%; *p* = 0.008), and by day 14 1.6-fold higher (4.4% vs. 2.8%, *p* = 0.02). Additionally, after 14 days, the proportion of CD4^+^CD25^+^ cells exceeded the control values by 1.8 times (4.4% vs. 2.5%; *p* = 0.025), which indicates a stable immunocorrective effect of MXF-22 aimed at restoring regulatory T cells. Moreover, treatment with MXF-22 had a more pronounced effect by 14 day than treatment with Polyoxidonium (4.4% vs. 2.1%, *p* = 0.005). Thus, the use of MXF-22 promotes the restoration and prolonged activation of regulatory T cells in acute pneumonia, which confirms its pronounced immunomodulatory potential.

After analyzing the CD4^+^CD25^+^ lymphocyte subpopulation, the content of CD4^+^FoxP3^+^ cells, the most specific representatives of regulatory T cells, was assessed. The expression of the FoxP3 transcription factor is a key marker of Treg functional activity, providing suppression of excessive inflammatory response and maintenance of immune homeostasis. The change in the proportion of CD4^+^ FoxP3^+^ cells makes it possible to more accurately characterize the degree of activation and regulatory potential of the immune system in acute pneumonia and under the influence of the studied drugs.

**Figure 4:**
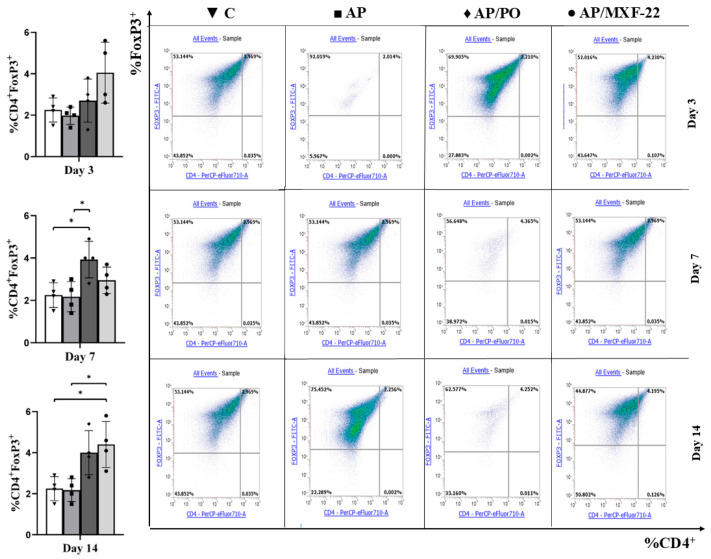
The effect of Polyoxidonium and the new synthetic compound MXF-22 on CD4^+^FoxP3^+^ lymphocyte count in acute pneumonia. After splenocyte isolation, the cells were stained with antibodies conjugated with fluorochromes, followed by analysis by flow cytometry. The figure shows dot diagrams (days 3, 7, 14) reflecting the distribution of CD4^+^FoxP3 cells; bar charts show the average values of the proportion of CD4^+^FoxP3^+^ lymphocytes (%) in the rat spleen on the 3rd, 7th, and 14th days of the experiment. Groups: **C**—control; **AP**—acute pneumonia without treatment; **AP/PO**—Polyoxidonium treatment; **AP/MXF-22**—MXF-22 treatment. Statistics were performed using two-way ANOVA, with multiple comparisons corrected with Tukey’s method. All data is presented as an average value ± standard deviation (*n* = 6). * *p* < 0.05.

When studying a subpopulation of CD4^+^ FoxP3^+^ lymphocytes, it was found that in AP group, the content of CD4^+^ FoxP3^+^ cells did not significantly differ from the control and amounted to 2.0–2.2% at all follow-up periods, which indicates the absence of pronounced spontaneous activation of the regulatory link of immunity during inflammation (Figure 4).

A statistically significant increase in the proportion of CD4^+^ FoxP3^+^ lymphocytes was observed following treatment with Polyoxidonium. By day 7, the value increased to 3.0%, exceeding both the control group (*p* = 0.045) and the acute pneumonia group (*p* = 0.05). Treatment with MXF-22 resulted in a more pronounced and steady increase in CD4^+^ FoxP3^+^ lymphocytes levels. After 14 days, the indicator reached 4.0%, statistically significantly exceeding the control (*p* = 0.048). After 7 days, the value remained elevated (3.0%), and by the 14th day it increased to 4.4%, which was significantly higher than both the control (*p* = 0.048) and acute pneumonia groups (*p* = 0.047).

Thus, similar to Polyoxidonium, MXF-22 promotes activation of regulatory T cells; however, it induces a more pronounced and prolonged increase in the content of CD4^+^ FoxP3^+^ lymphocytes. These findings confirm the high immunomodulatory potential of the compound in the correction of inflammatory disturbances associated with acute pneumonia.

The obtained data on T-lymphocytes dynamics in acute pneumonia provide the basis for further analysis of the relationship of these cells with the cytokine profile, particularly IL-4 and TGF-β levels. Subsequent studies are aimed at clarifying these correlations, which will allow a more comprehensive assessment of the role of regulatory T cells in shaping the anti-inflammatory response induced by the action of MXF-22 and Polyoxidonium.

**Figure 5:**
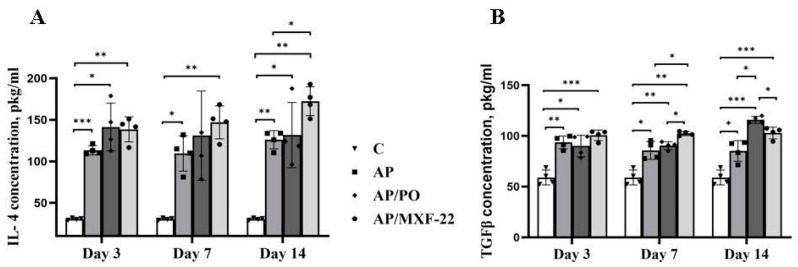
Effects of Polyoxidonium and the novel synthetic compound MXF-22 on the levels of pro- and anti-inflammatory cytokines in acute pneumonia. (**A**) Levels of cytokine IL-4; (**B**) Levels of cytokine TGF-β. Bar charts illustrate serum cytokine concentrations in rats on days 3, 7, and 14 of the experiment. Groups: **C**—control; **AP**—acute pneumonia without treatment; **AP/PO**—Polyoxidonium treatment; **AP/MXF-22**—MXF-22 treatment. Statistics were performed using two-way ANOVA, with multiple comparisons corrected with Tukey’s method. All data is presented as an average value ± standard deviation (*n* = 6). * *p* < 0.05; ** *p* < 0.01; *** *p* < 0.001.

The observed changes in the content of CD4^+^FoxP3^+^ lymphocytes in acute pneumonia suggests a possible association with the activity of anti-inflammatory cytokines, primarily interleukin-4. To clarify the mechanism of the immunomodulatory effect of MXF-22 and Polyoxidonium, aimed at restoring the balance of the Th and Treg components of the immune response, a study was conducted on the ratio of IL-4 levels to regulatory T cells (Figure 5A).

In acute pneumonia, the concentration of IL-4 in the blood serum increased significantly compared with the control (Figure 5A). After 3 days, the cytokine level increased approximately 3.7 times (113.3 vs. 30.5 pg/mL; *p* = 0.0001), 3.6 times after 7 days (*p* = 0.01), and 4.1 times by day 14 (*p* = 0.001), indicating sustained activation of IL-4 production in response to the inflammatory process. Polyoxidonium treatment was accompanied by a further increase in IL-4 levels: 4.6-fold above control on day 3 (*p* = 0.01), 4.3-fold on day 7 (*p* = 0.05), and 4.3-fold on day 14 (*p* = 0.04). However, compared with the AP group, no statistically significant difference was observed. MXF-22 led to the most pronounced increase in IL-4 levels. After 3 days, IL-4 levels were 4.5-fold higher than control (138.5 vs. 30.5 pg/mL; *p* = 0.002) and after 14 days 5.7-fold higher than control (172.4 vs. 30.5 pg/mL; *p* = 0.001). By day 14, IL-4 levels were 5.6-fold higher than in the AP group (*p* = 0.02). These data propose that MXF-22 enhances the production of IL-4 to a greater extent than Polyoxidonium, contributing to the activation of the anti-inflammatory immune response.

An increase in IL-4 levels in the treatment of acute pneumonia indicates activation of the anti-inflammatory immune response and a possible strengthening of regulatory mechanisms. For a more complete understanding of cytokine interaction at this stage, it is important to consider the role of TGF-β, which, unlike IL-4, provides not only suppression of inflammation, but also maintenance of immunological tolerance due to activation and differentiation of regulatory T cells.

TGF-β plays a key role in the regulation of inflammation and tissue repair. In acute pneumonia, an increase in TGF-β level reflects the activation of anti-inflammatory and regenerative mechanisms, as well as the differentiation of regulatory T cells, which makes TGF-β an important indicator of the effectiveness of immunocorrective therapy. In rats with acute pneumonia, serum TGF-β concentrations were higher compared with the control group (Figure 5B). On day 3 of the acute pneumonia development, the concentration of TGF-β increased approximately 1.6-fold (93.7 vs. 59.1 pg/mL; *p* = 0.002), reflecting activation of compensatory mechanisms aimed at limiting inflammation. On days 7 and 14, the level of TGF-β remained steadily elevated—1.4-fold above control (*p* = 0.01 and *p* = 0.03, respectively), which indicates the preservation of regulatory potential in the inflammatory response. Treatment with Polyoxidonium demonstrated a similar pattern: the concentration of TGF-β increased by approximately 1.5-fold on days 3 and 7 compared to control (90.2 and 90.5 vs. 59.1 pg/mL; *p* = 0.02 and *p* = 0.003, respectively), and doubled by day 14 (116 vs. 59.1 pg/mL; *p* = 0.0004). Also, statistically significant differences were observed in the AP/PO group compared with the AP group on day 14 (*p* = 0.02), proposing activation of reparative processes and enhancement of anti-inflammatory responses.

Treatment with MXF-22 was associated with the most pronounced and sustained increase in the level of TGF-β. 3 days post pneumonia development, its concentration rose 1.7-fold compared with the control group (100.7 vs. 59.1 pg/mL; *p* = 0.0006), and a similar 1.7-fold elevation was observed on day 7 (102.5 vs. 59.1 pg/mL; *p* = 0.002). Serum TGF-β levels remained significantly elevated by day 14 (*p* = 0.0005). When compared with the acute pneumonia group, after 7 days, TGF-β levels increased by about 19% in the group receiving MXF-22 (102.5 vs. 85.8 pg/mL; *p* = 0.05), suggesting a more pronounced activation of anti-inflammatory mechanisms during the early phase of treatment. These findings indicate that MXF-22 contributes to the maintenance of stable TGF-β levels, which is probably due to the activation of regulatory T cells and enhancement of lung tissue repair processes during the resolution phase of inflammation.

Thus, treatment with MXF-22 demonstrated its ability to stimulate both anti-inflammatory and regenerative mechanisms of the immune response.

## 4. Discussion

Acute respiratory infections trigger an inflammatory immune response aimed at eliminating the pathogen. However, excessive inflammation causes tissue damage and disrupts lung function. CD4^+^CD25^+^FoxP3^+^ regulatory T cells interact with cells of both the innate and adaptive immune systems to limit acute lung inflammation and promote its resolution. Tregs also provide tissue protection and coordinate the repair of lung tissue, contributing to the return of homeostatic lung function [36,37,38].

The study showed that the development of acute pneumonia is associated with marked changes in the regulatory mechanisms of the immune response, especially for CD4^+^FoxP3^+^ T cells and cytokines involved in maintaining immune homeostasis. In animals with the acute pneumonia model, there was a decrease in the number of CD4^+^ FoxP3^+^ cells and a violation of the ratio of anti-inflammatory cytokines, including IL-4 and TGF-β. These changes reflect a weakening of Treg suppressive activity, which contributes to an increased inflammatory cascade and damage to lung tissue [39]. Infusion of CD4^+^ FoxP3^+^ Treg 24 h after administration of LPS promoted accelerated resolution of inflammation and restoration of lung tissue in Rag mice-1^−^/^−^ [40]. On the contrary, Treg depletion in wild-type animals led to a slowdown in reparative processes, which confirms their key role in controlling the inflammatory response. Treg transplantation was accompanied by a decrease in the level of proinflammatory cytokines, an increase in the content of TGF-β, and increased neutrophil apoptosis. At the same time, blocking TGF-β completely eliminated the Treg-mediated effect, emphasizing the dependence of recovery processes on TGF-β signaling of the ability of Treg cells to promote the restoration of lung tissue, which indicates that regulatory T cells are actively involved in modulating the innate immune response in the process of resolving inflammation and lung repair [40].

Previously published studies have shown that piperidine derivatives exhibit antioxidant and DNA-binding activity [41], as well as antisecretory, gastroprotective [42], and anticancer [43] effects. In our studies treatment with MXF-22 demonstrated a significant increase in the level of CD4^+^ FoxP3^+^ cells, which suggests the restoration of the regulatory mechanisms of the immunity. An increase in the expression of FoxP3^+^, a key transcription factor of Treg, confirms the activation of mechanisms of immune tolerance and anti-inflammatory control. One of the proposed mechanisms explaining our findings could be an indirect effect through the action of MXF-22 on the antigen-presenting cells (dendritic cells, macrophages) and their expression of surface molecules that favor the induction of Tregs. Previous in vitro studies demonstrated the ability of small heterocycle molecules to induce APC phenotype shift through the activation of the c-Jun N-terminal kinases and the suppression of extracellular signal-regulated kinases that would favor tolerogenic outcomes and reduce effector T cell priming [44].

At the same time, increased levels of IL-4 and TGF-β were noted, reflecting a switch in the immune response towards an anti-inflammatory profile and activation of repair processes.

The increased production of TGF-β in the treatment of MXF-22 is especially significant compared with the AP group without treatment. This cytokine, combined with the increased activity of FoxP3 cells, helps to suppress excessive inflammatory response and maintain homeostatic balance in lung tissue. The possible molecular mechanism of action is through the interaction of MXP-22 with Aryl hydrocarbon receptor (AHR). AHR is a ligand-dependent transcription factor responsible for recognizing xenobiotics and regulating immune and barrier functions. It was found that some piperidine derivatives could modulate AHR activity in vitro models [45] and ligand-specific activation of the AHR itself could promote FoxP3^+^ Treg differentiation [46]. Moreover, it was established that the AHR signaling pathway is functionally interconnected with the TGF-β/Smad cascade. Specifically, AHR activation can enhance TGF-β signaling by stabilizing Smad2/3 transcriptional complexes, whereas TGF-β, in turn, can increase the expression of components involved in AHR activation. This bidirectional regulation creates favorable conditions for the induction of a tolerogenic phenotype in immune cells and the subsequent development of Treg-mediated immune regulation [47,48].

Thus, MXF-22 showed a pronounced immunomodulatory effect aimed at restoring the activity of CD4^+^FoxP3^+^ Treg cells and normalizing the production of key anti-inflammatory cytokines. These effects underlie its ability to limit hyperinflammation and accelerate reparative processes in acute pneumonia.

In our previous study, we investigated the effects of the structurally related piperidine derivative EPPPβCD using a model of aseptic inflammation [23]. This model was applied to examine the mechanisms of inflammation chronification under xenobiotic exposure [28], as heavy metals produce pronounced immunosuppression. The use of EPPPβCD markedly accelerated inflammation resolution by inhibiting the Treg response. A comparison of the two models demonstrates that the compounds exert their effects through different mechanisms, as the underlying immunopathological conditions are opposite in nature: metal-induced exposure is characterized by immunosuppression and an excess of Treg cells [49,50] whereas acute pneumonia is accompanied by hyperinflammation and a deficit of regulatory T cells [51,52,53]. EPPPβCD restores suppressed immunity by reducing excessive regulatory signaling, while MXF-22 re-establishes the physiological level of Treg-mediated inflammatory control. Despite these mechanistic differences, both compounds ultimately lead to the restoration of immune balance and effective resolution of the inflammatory response, adapting to the specific features of the initial immune disturbances.

At the same time, our research continues aimed at a more detailed study of the mechanisms of action of MXF-22 and its therapeutic potential.

## 5. Conclusions

In summary, the target propionate derivative **EPP-pr** was efficiently synthesized in two steps from 1-(2-ethoxypropyl)-4-oxopiperidine, and its structure—along with that of the intermediate **EPP-ol**—was confirmed by FT-IR, ^1^H/^13^C NMR, and 2D NMR spectroscopy. Complexation of EPP-pr with β-CD afforded the stable inclusion complex **MXF-22** in high yield, and its formation was supported by characteristic FT-IR features, diagnostic chemical-shift perturbations in the ^1^H NMR spectra, and the disappearance of the free **EPP-pr** spot in TLC analysis. These data collectively confirm successful encapsulation of the propionate derivative within the β-CD cavity, improving its physicochemical stability and supporting its potential for further biomedical evaluation.

MXF-22 has a pronounced immunomodulatory effect, contributing to the restoration of the function of CD4^+^ FoxP3^+^ regulatory T cells in acute pneumonia.

The drug normalizes the production of key anti-inflammatory cytokines, which controls the hyperinflammatory response and prevents excessive damage to lung tissue.

The results obtained emphasize the prospects of MXF-22 as an immunomodulator in acute pneumonia, while further research is aimed at clarifying the molecular mechanisms of its action.

## Figures and Tables

**Table 1:** Antibodies and dyes for immunophenotyping of rat spleen subcellular populations treated with PO or MXF-22.

Category Code	Antibody	Fluorochrome	Manufacturer	Clone
46-0040-82	CD4	PerCP-eFluor™ 710	Invitogen, Thermo Fisher Scientific, Waltham, MA, USA	OX-35
17-0390-82	CD25	APC		OX-39
11-5773-82	FoxP3	FITC		FJK-16s

**Table 2:** ELISA Kits used in the experiment.

Cytokine	Kit	Sample Type	Manufacturer	Catalog No
IL-4	Rat IL-4 ELISA Kit	Serum	Invitrogen, Thermo Fisher Scientific, Waltham, MA, USA	BMS628-BMS628TEN
TGF-β	Rat TGFβ ELISA Kit	Serum		BMS623-2 & BMS623-3TEN

## Data Availability

The data used to support the findings in the study are available in the Supplementary Materials. Other data underlying this article will be shared upon reasonable written request to the corresponding author after publication.

## Supplementary Materials

The following supporting information can be downloaded at: https://www.mdpi.com/article/10.3390/biomedicines13123017/s1, Figure S1: IR (KBr, ν, cm^−1^) spectrum of **EPP-ol**; Figure S2: IR (KBr, ν, cm^−1^) spectrum of **EPP-pr**; Figure S3: IR (KBr, ν, cm^−1^) spectrum of **MXF-22**; Figure S4: IR (KBr, ν, cm^−1^) spectrum of β-CD; Figure S5: The ^1^H NMR spectrum of **EPP-ol** in CDCl_3_; Figure S6: The ^13^C NMR spectrum of **EPP-ol** in CDCl_3_; Figure S7: The correlation scheme in the COSY (a), HMQC (b), and HMBC (c) spectra of **EPP-ol**; Figure S8: The COSY spectrum of **EPP-ol**; Figure S9: The HMQC spectrum of **EPP-ol**; Figure S10: The HMBC spectrum of **EPP-ol**; Figure S11: The ^1^H NMR spectrum of **EPP-pr** in CDCl_3_; Figure S12: The ^13^C NMR spectrum of **EPP-pr** in CDCl_3_; Figure S13: The correlation scheme in the COSY (a), HMQC (b), and HMBC (c) spectra of **EPP-pr**; Figure S14: The COSY spectrum of **EPP-pr**; Figure S15: The HMQC spectrum of **EPP-pr**; Figure S16: The HMBC spectrum of **EPP-pr**; Figure S17: The ^1^H NMR spectrum of MXF-2 in DMSO-d6; Figure S18: The ^13^C NMR spectrum of MXF-2 in DMSO-d6; Figure S19: The COSY spectrum of **MXF-22**; Figure S20: The HMQC spectrum of **MXF-22**; Figure S21: The HMBC spectrum of **MXF-22**; Figure S22: The NOESY spectrum of **MXF-22**; Figure S23: The ROESY spectrum of **MXF-22**; Figure S24: The TOCSY spectrum of **MXF-22**; Figure S25: Some Numbered Protons of the β-CD Glucopyranose Moiety; Table S1: Chemical shifts of the ^1^H and ^13^C nuclei of EPP-pr and β-cyclodextrin in their free states (δ_0_) and in the inclusion complex—**MXF-22** (δ).

## Abbreviations

The following abbreviations are used in this manuscript:

AHR	Aryl hydrocarbon receptor
AP	Acute Pneumonia
APC	Antigen-Presenting Cells
BSA	Bovine serum albumin
β-CD	β-cyclodextrin
C	Control
CD25	Cluster of differentiation 25
CD4	Cluster of differentiation 4
DMSO	d_6_-dimethyl sulfoxide
ELISA	Enzyme-Linked Immunosorbent Assay
EPP-ol	1-(3-Ethoxypropyl)-4-(pent-1-yn-1-yl)piperidin-4-ol
EPP-pr	1-(3-Ethoxypropyl)-4-(pent-1-yn-1-yl)piperidin-4-yl Propionate
FMO	Fluorescence-minus-one
FoxP3	Forkhead Box Protein P3
FT-IR	Fourier Transform Infrared spectroscopy
IL-4	Interleukin-4
IR	Infrared spectroscopy
MXF-22	Complex of 1-(2-Ethoxypropyl)-4-(pent-1-yn-1-yl)piperidin-4-yl Propionate with β-Cyclodextrin
NF-κB	Nuclear Factor-kappa B
NMR	Nuclear Magnetic Resonance
OA	Oleic-acid
PO	Polyoxidonium
Smad	SMAD family proteins (homologs of Sma and Mad)
TGF-β	Transforming growth factor-β
Th	T helper cells
TLC	Thin-layer chromatography
Treg	Regulatory T cells
