# Supplementary material for: Immunomodulatory Effects of a New Ethynylpiperidine Derivative: Enhancement of CD4+FoxP3+ Regulatory T Cells in Experimental Acute Lung Injury
Source: Biomedicines. 2025 Dec 9;13(12):3017. doi: 10.3390/biomedicines13123017 (PMC12730231; doi:10.3390/biomedicines13123017)
Supplement: Supplementary file 1 [file biomedicines-13-03017-s001.zip › biomedicines-3991068-supplementary.pdf]

SUPPLEMENTARY MATERIALS

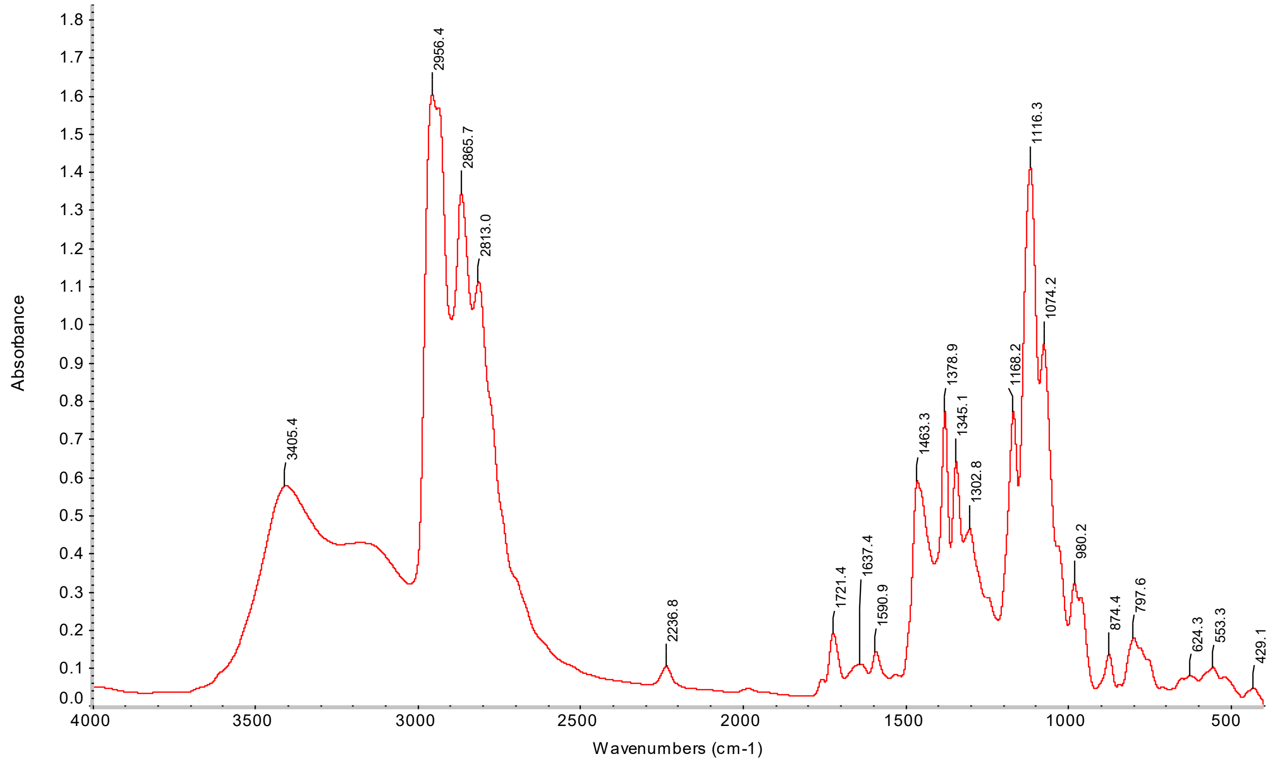

Figure S1. IR (KBr, ν, cm^{-1}) spectrum of **EPP-ol**

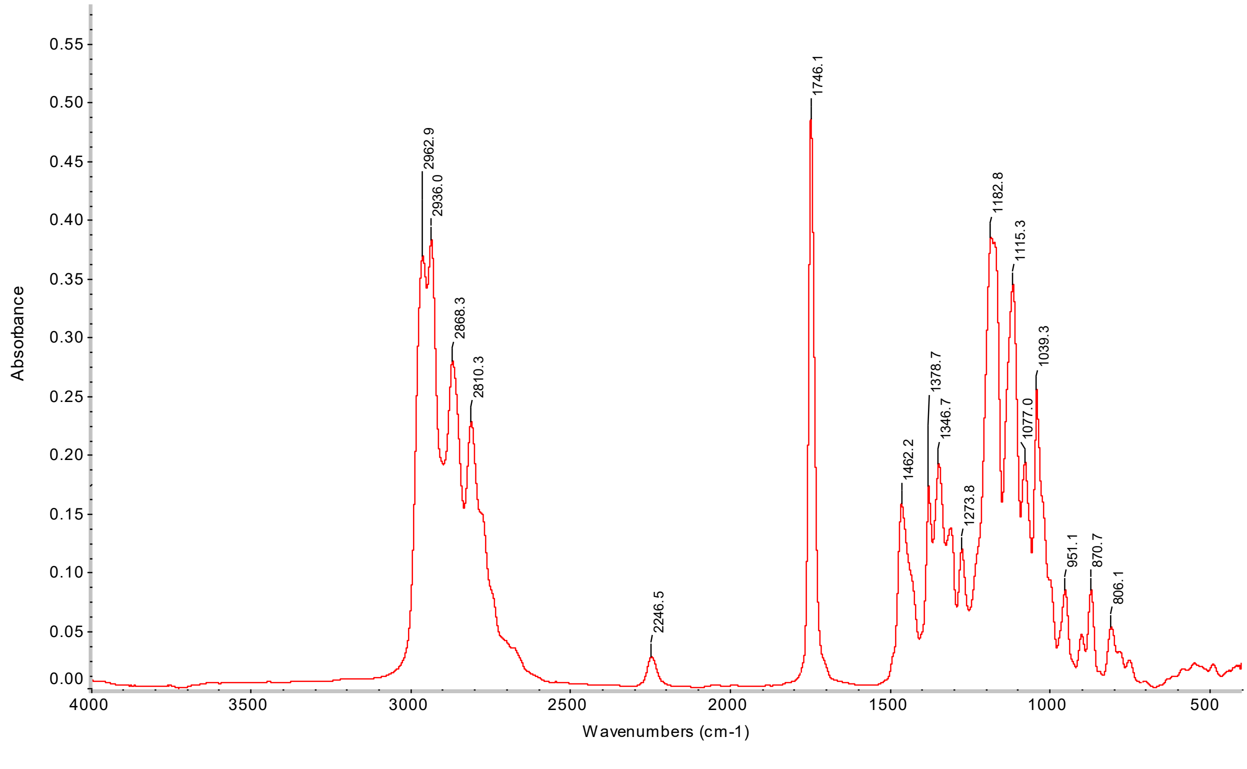

Figure S2. IR (KBr, ν, cm^{-1}) spectrum of **EPP-pr**

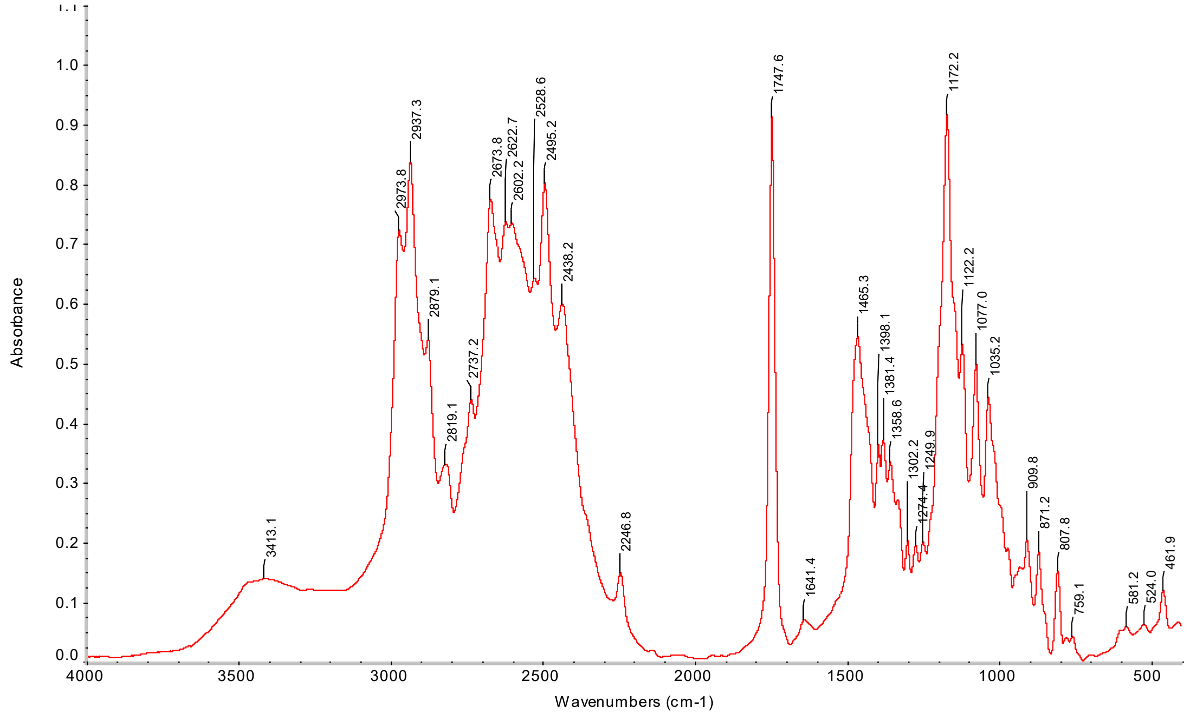

Figure S3. IR (KBr, ν, cm^{-1}) spectrum of **MXF-22**

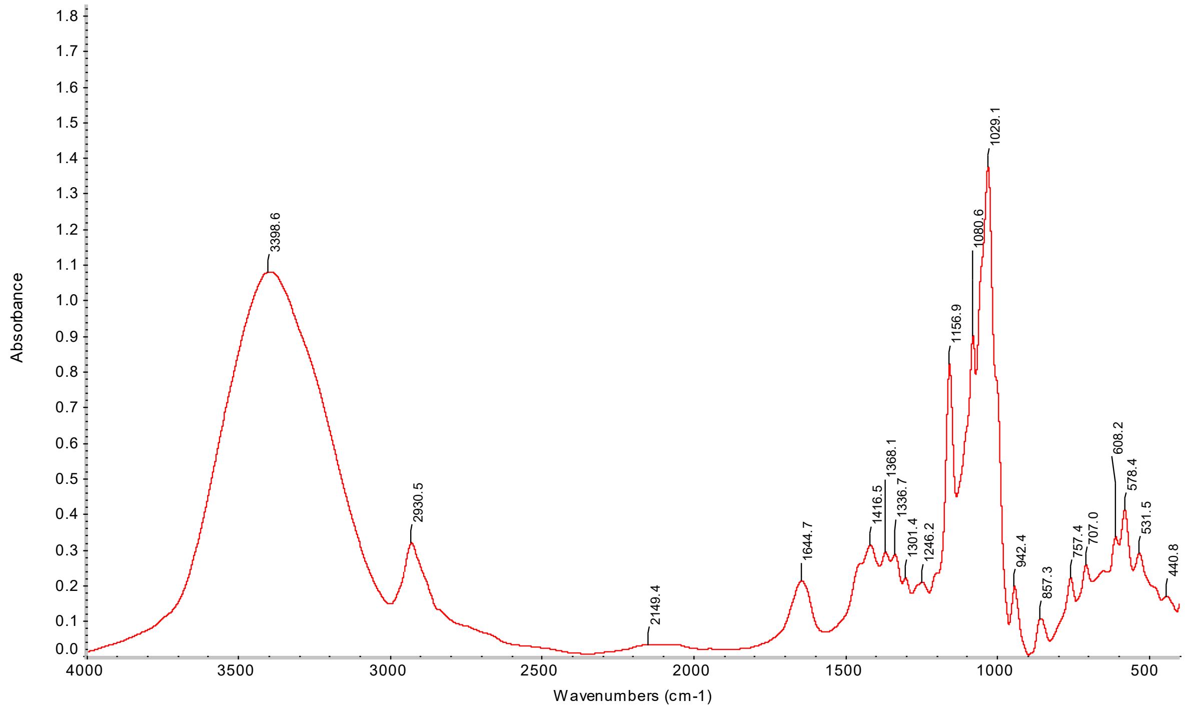

Figure S4. IR (KBr, ν, cm^{-1}) spectrum of β-CD

Analysis of the NMR spectra of EPP-ol in $CDCl_3$

In the ^1H NMR spectrum of **EPP-ol**, the methyl protons H-18,18,18 of the pentynyl fragment appear as a three-proton triplet at 0.91 ppm (^3J = 7.4 Hz). The methylene protons H-17,17 and H-16,16 resonate as a two-proton multiplet at 1.40–1.48 ppm and a triplet at 2.11 ppm (^3J = 6.8 Hz), respectively. The methyl protons H-12,12,12 of the N-ethoxypropyl group give a triplet at 1.12 ppm (^3J = 7.2 Hz). The methylene protons H-8,8 appear together with the piperidine protons H-3ax,5ax and H-3eq,5eq as a six-proton multiplet at 1.64–1.83 ppm. Protons H-7,7 resonate together with H-2ax,6ax as a four-proton multiplet at 2.27–2.38 ppm. The piperidine protons H-2eq,6eq appear as a broad two-proton singlet at 2.61 ppm. The hydroxyl proton H-13 is observed as a broad one-proton singlet at 3.05 ppm. The methylene protons H-11,11 and H-9,9 of the N-ethoxypropyl fragment give a four-proton multiplet at 3.36–3.42 ppm.

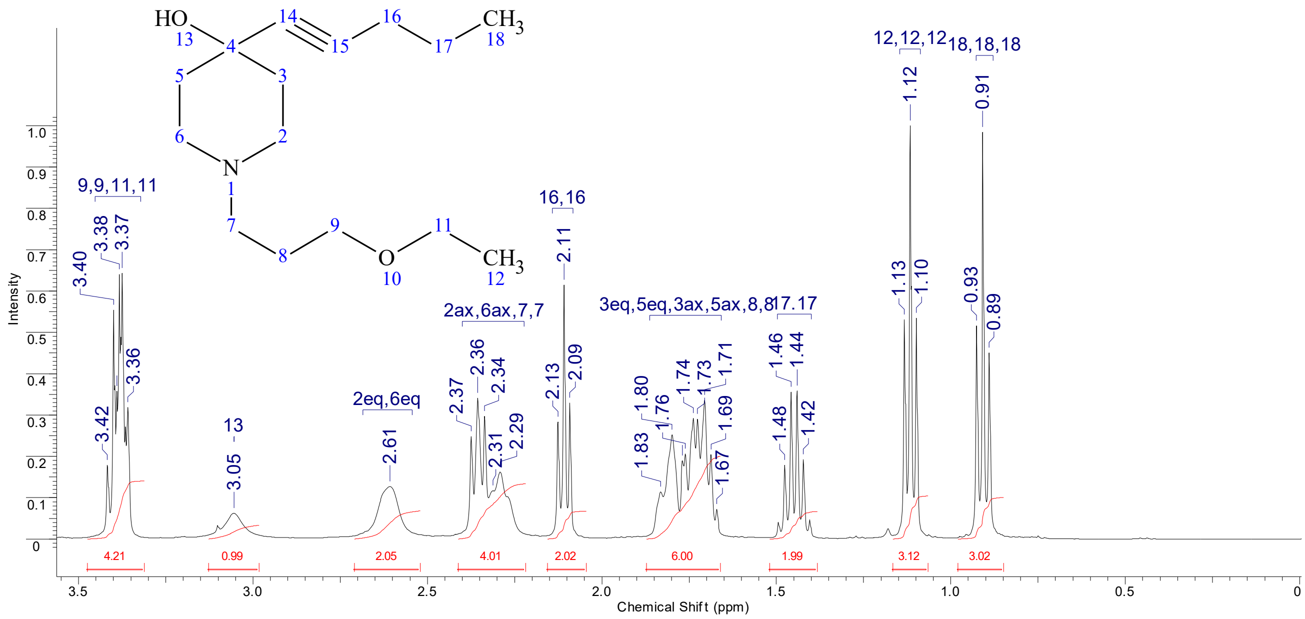

Figure S5. The ^1H NMR spectrum of **EPP-ol** in $CDCl_3$

In the ^{13}C NMR spectrum, signals of the pentynyl fragment appear at 13.55 (C-18), 20.66 (C-16), 22.21 (C-17), 83.88 (C-15), and 85.09 (C-14) ppm. The N-ethoxypropyl fragment carbons resonate at 15.24 (C-12), 27.30 (C-8), 55.16 (C-7), 66.11 (C-11), and 69.13 (C-9) ppm. The piperidine ring carbons are observed at 39.32 (C-3,5), 50.50 (C-2,6), and 67.07 (C-4) ppm.

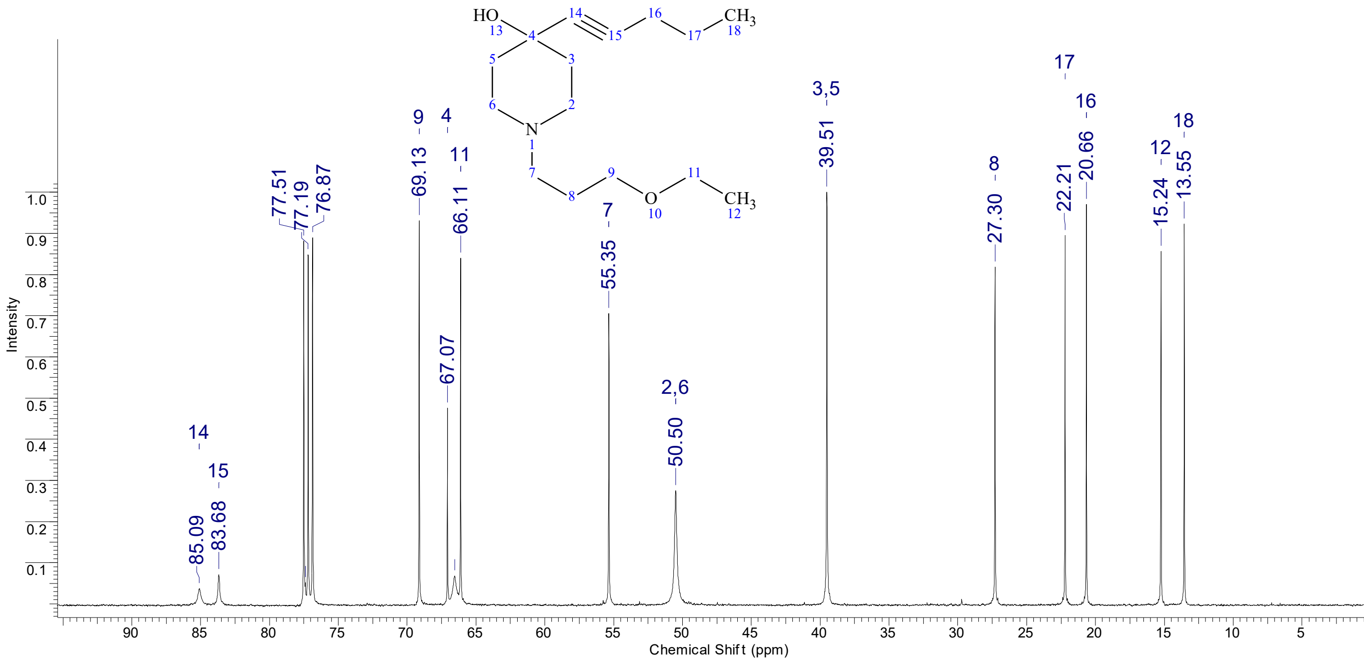

Figure S6. The ^{13}C NMR spectrum of **EPP-ol** in $CDCl_3$

The structure of EPP-ol was further confirmed by 2D NMR experiments, including COSY (^1H–^1H), HMQC (^1H–^{13}C), and HMBC (^1H–^{13}C), which established both homo- and heteronuclear spin–spin correlations.

COSY correlations were observed for the following pairs: H18→H17, H17→H16, H3eq,5eq→H2ax,6ax, H12→H11, H8→H7, H8→H9, H2ax,6ax→H2eq,6eq.

HMQC revealed one-bond ^1H–^{13}C correlations: H18→C18, H12→C12, H17→C17, H8→C8, H16→C16, H11→C11, H9→C9, H3ax,5ax→C3,5, H2ax,6ax→C2,6, and H2eq,6eq→C2,6.

HMBC demonstrated long-range correlations, including: H18→C17; H12→C11; H17→C18, C16, C14; H8→C7, C9; H16→C18, C17, C15; H2ax,6ax→C9; H9→C12; H11→C8, C7.

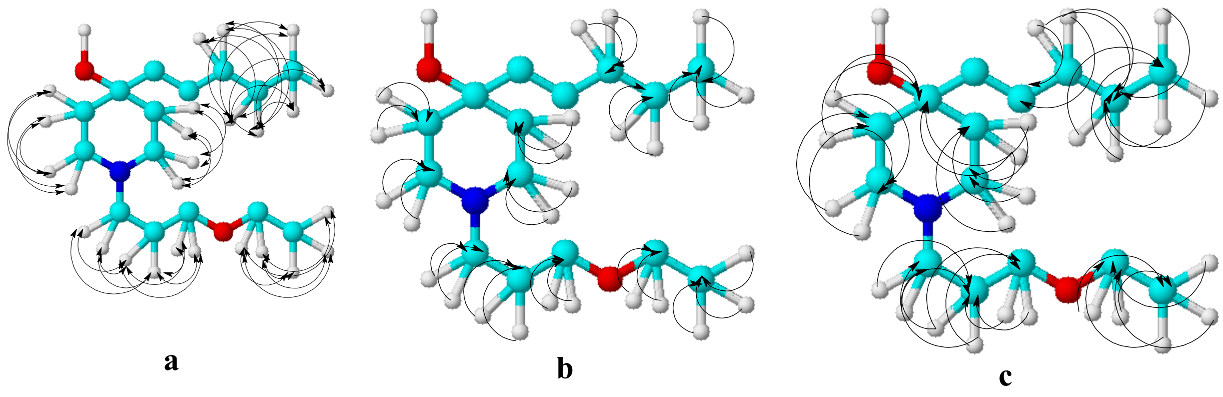

Figure S7. The correlation scheme in the COSY (**a**), HMQC (**b**), and HMBC (**c**) spectra of **EPP-ol**.

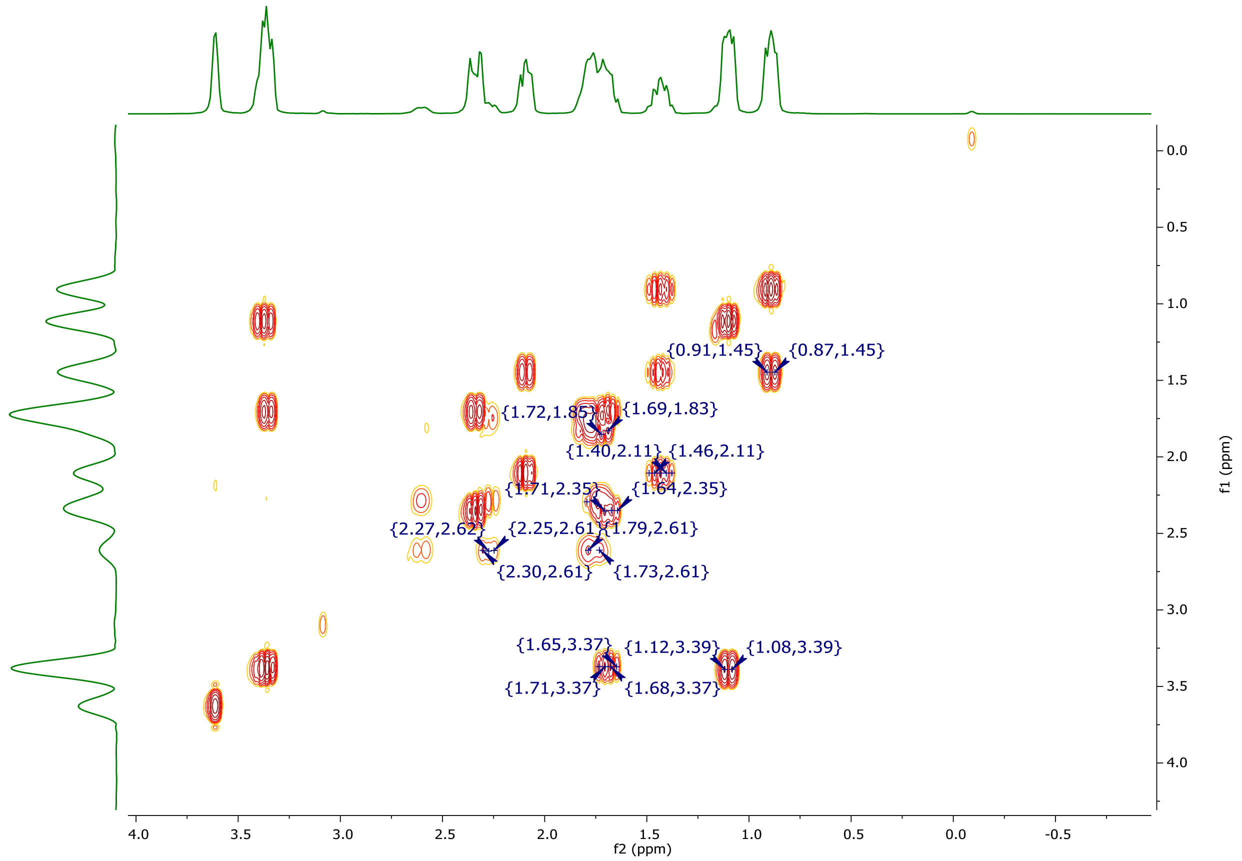

Figure S8. The COSY spectrum of **EPP-ol**

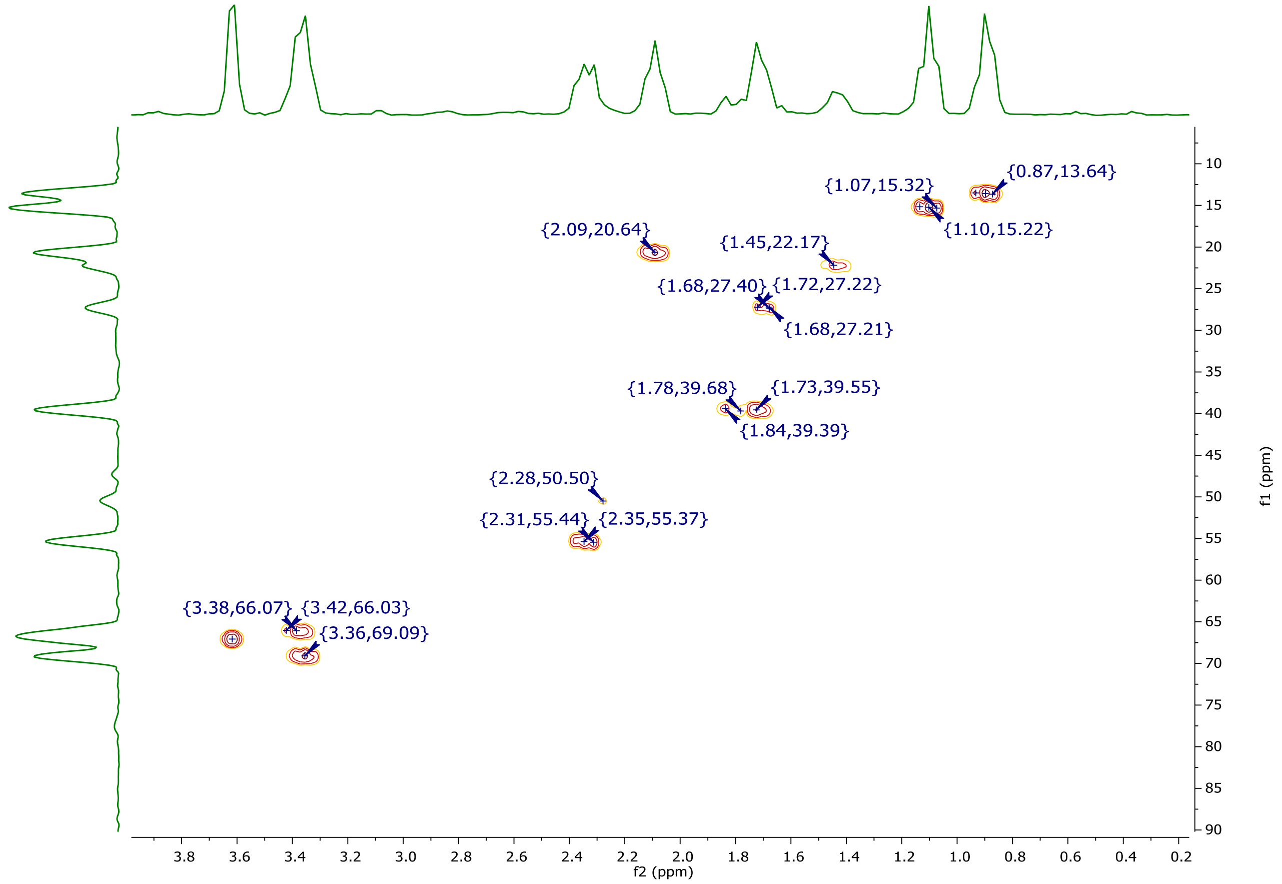

Figure S9. The HMQC spectrum of **EPP-ol**

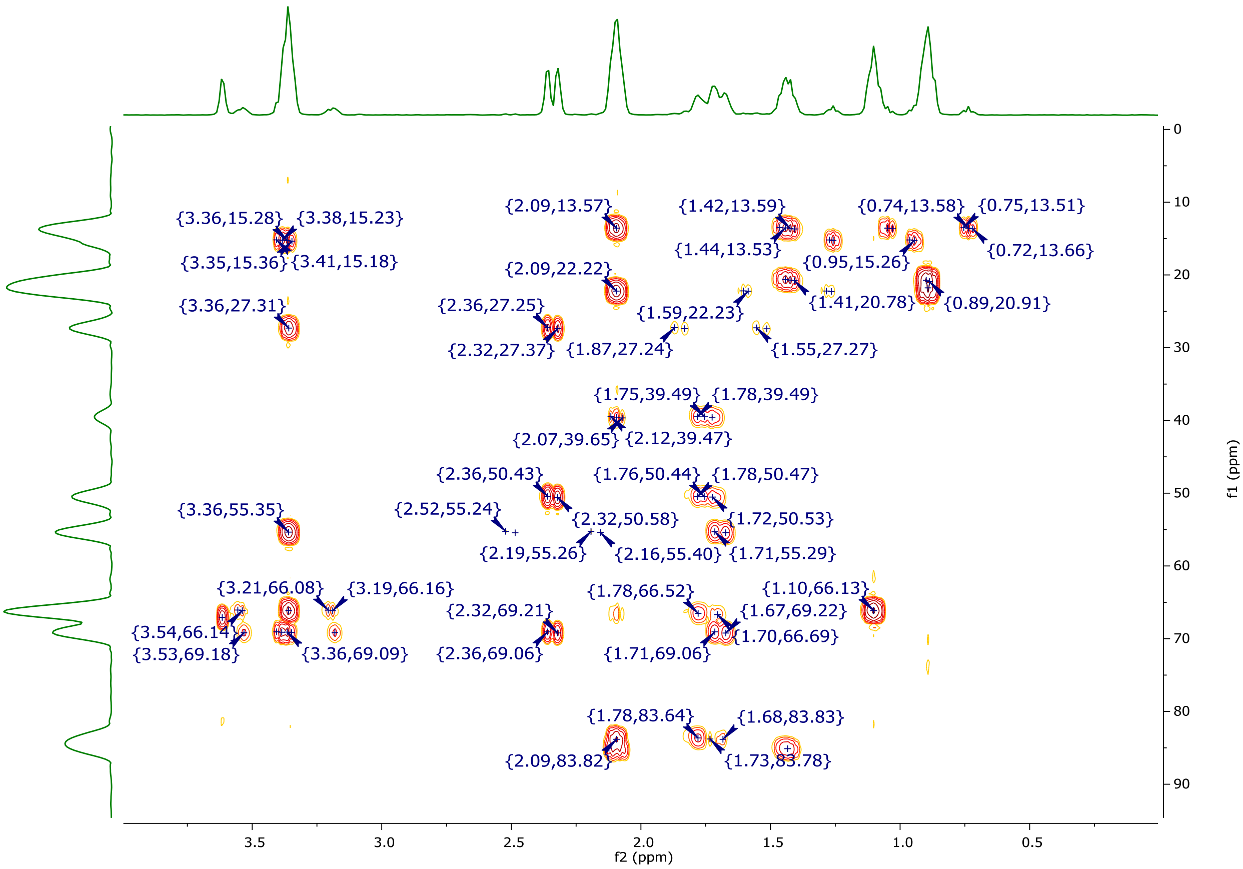

Figure S10. The HMBC spectrum of **EPP-ol**

Analysis of the NMR spectra of EPP-pr in $CDCl_3$

In the ^1H NMR spectrum of EPP-pr, the methyl protons H-20,20,20 of the pentenyl fragment appear as a three-proton multiplet at 0.88–0.93 ppm. The methylene protons H-19,19 resonate as a two-proton multiplet at 1.42–1.51 ppm, while protons H-18,18 appear together with the piperidine protons H-3eq,5eq as a four-proton multiplet at 2.10–2.16 ppm. The methyl protons H-22,22,22 of the propionyloxy group give a three-proton multiplet at 1.04–1.08 ppm, and the methylene protons H-21,21 resonate as a two-proton multiplet at 2.21–2.26 ppm. The methyl protons H-12,12,12 of the N-ethoxypropyl fragment give a three-proton multiplet at 1.11–1.15 ppm. The methylene protons H-8,8 appear as a two-proton multiplet at 1.66–1.73 ppm, while the methylene protons H-7,7 resonate together with the piperidine protons H-2ax,6ax as a four-proton multiplet at 2.33–2.39 ppm. The piperidine protons H-3ax,5ax and H-2eq,6eq appear as broadened two-proton singlets at 1.97 and 2.52 ppm, respectively. The methylene protons H-11,11 and H-9,9 of the N-ethoxypropyl fragment give a four-proton multiplet at 3.37–3.43 ppm.

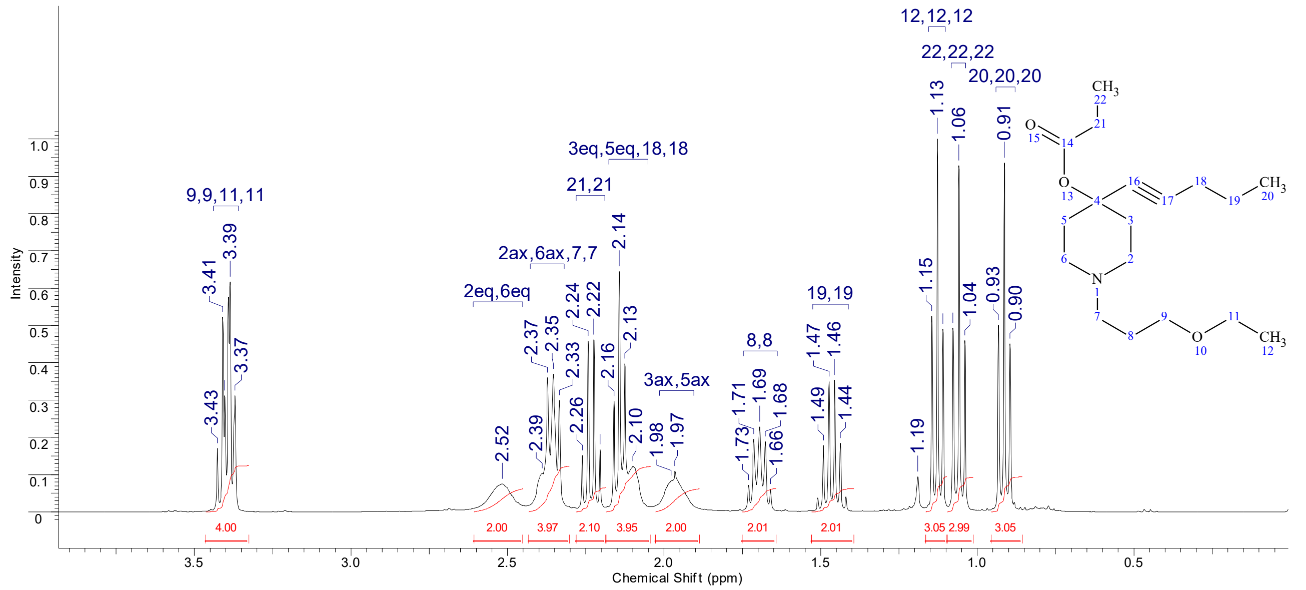

Figure S11. The ^{1}H NMR spectrum of **EPP-pr** in $CDCl_3$

In the ^{13}C NMR spectrum, signals of the pentenyl fragment appear at 13.48 (C-20), 20.57 (C-18), 22.13 (C-19), 79.72 (C-16), and 87.22 (C-17) ppm. The N-ethoxypropyl fragment carbons resonate at 15.27 (C-12), 27.50 (C-8), 55.35 (C-7), 66.16 (C-11), and 69.05 (C-9) ppm. The piperidine ring carbons are observed at 37.02 (C-3,5), 50.04 (C-2,6), and 73.70 (C-4) ppm. The propionyloxy group carbons appear at 9.16 (C-22), 28.60 (C-21), and 172.77 (C-14) ppm.

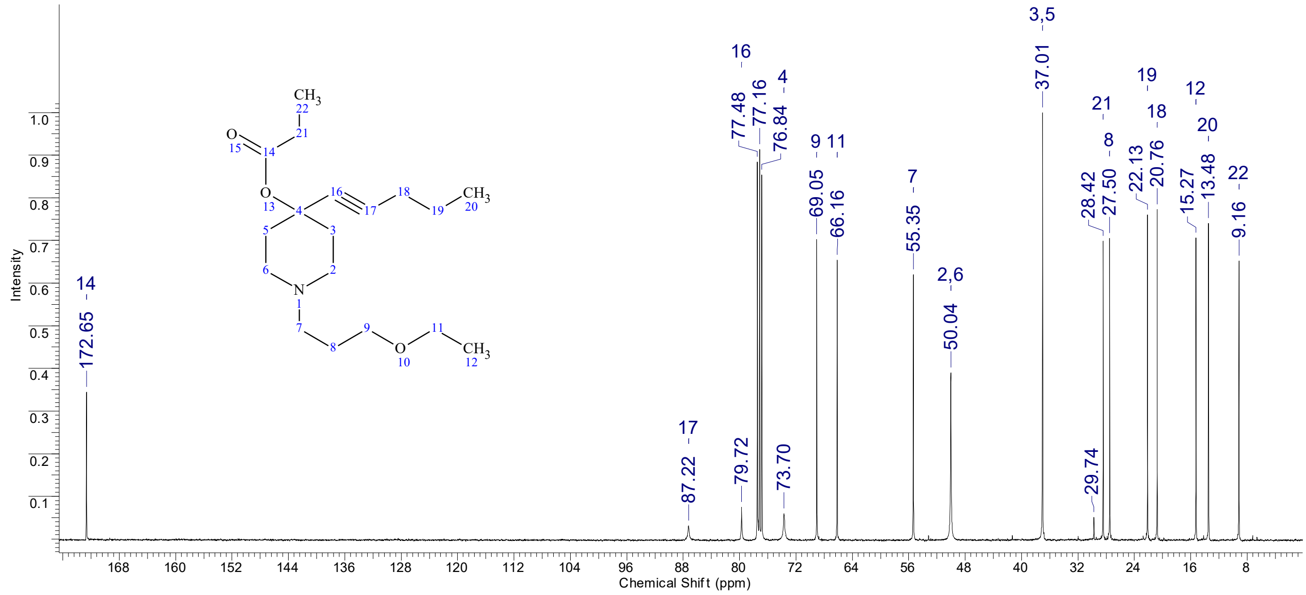

Figure S12. The ^{13}C NMR spectrum of **EPP-pr** in $CDCl_3$

The structure of EPP-pr was further confirmed by 2D NMR experiments, including COSY (^{1}H–^{1}H), HMQC (^{1}H–^{13}C), and HMBC (^{1}H–^{13}C), which established both homo- and heteronuclear spin–spin correlations.

COSY correlations were observed for the following pairs: H20→H19, H19→H18, H22→H21, H8→H7, H3ax,5ax→H3eq,5eq, H12→H11, and H8→H9.

HMQC revealed one-bond ^{1}H–^{13}C correlations: H22→C22, H20→C20, H18→C18, H12→C12, H19→C19, H21→C21, H8→C8, H7→C7, H11→C11, H9→C9, H3ax,5ax→C3,5, H3eq,5eq→C3,5, H2ax,6ax→C2,6, and H2eq,6eq→C2,6.

HMBC demonstrated long-range correlations, including: H20→C18; H22→C21, C14; H12→C11; H19→C20, C18, C17; H8→C7, C9; H18→C20, C19, C16, C17; H21→C22, C14; H7→C9; H2ax,6ax→C3,5; H9→C8, C7, C11; and H11→C9, C12.

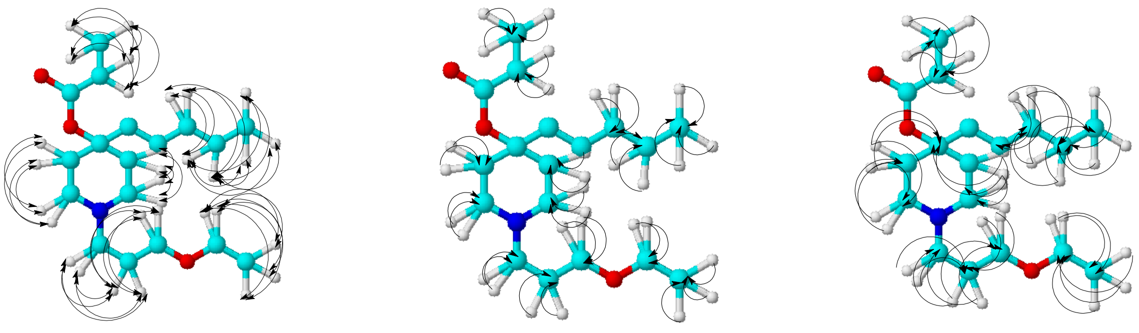

a b c

Figure S13. The correlation scheme in the COSY (**a**), HMQC (**b**), and HMBC (**c**) spectra of **EPP-pr**

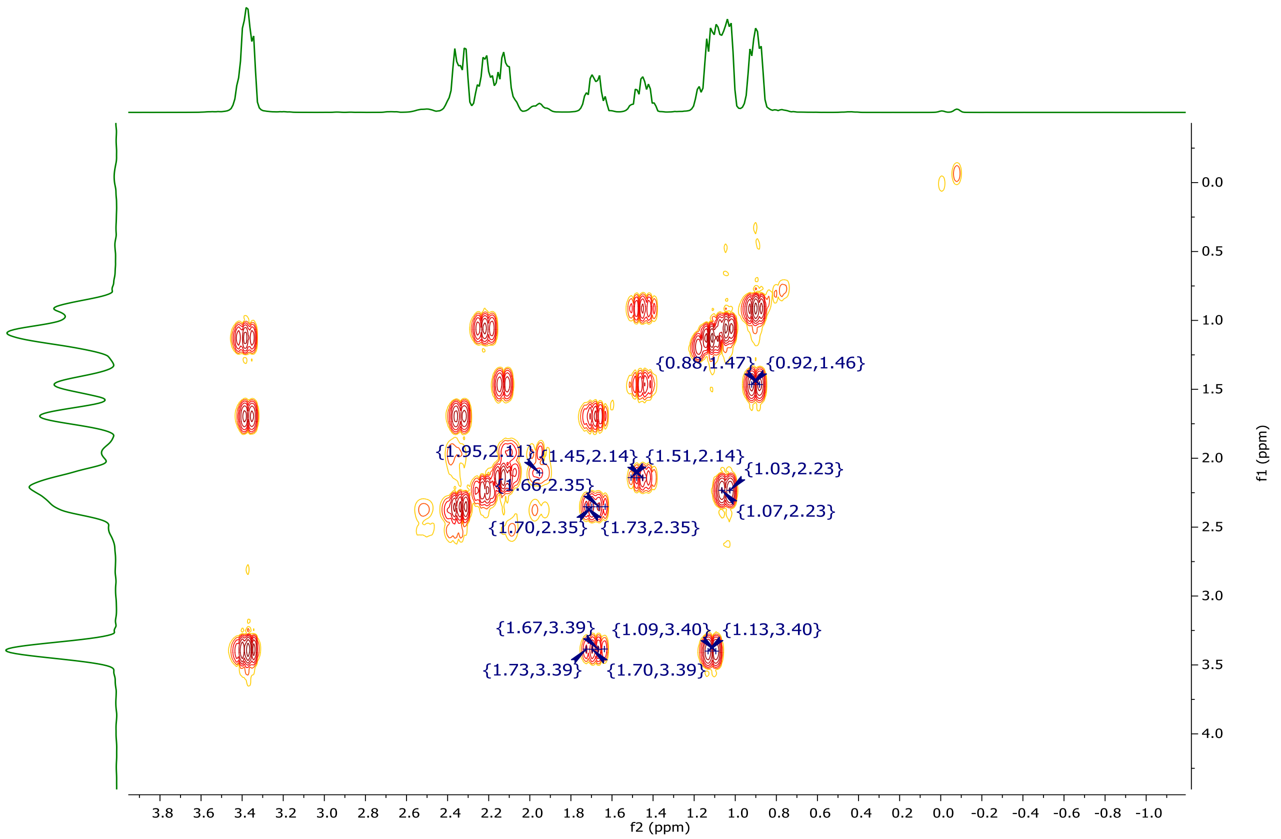

Figure S14. The COSY spectrum of **EPP-pr**

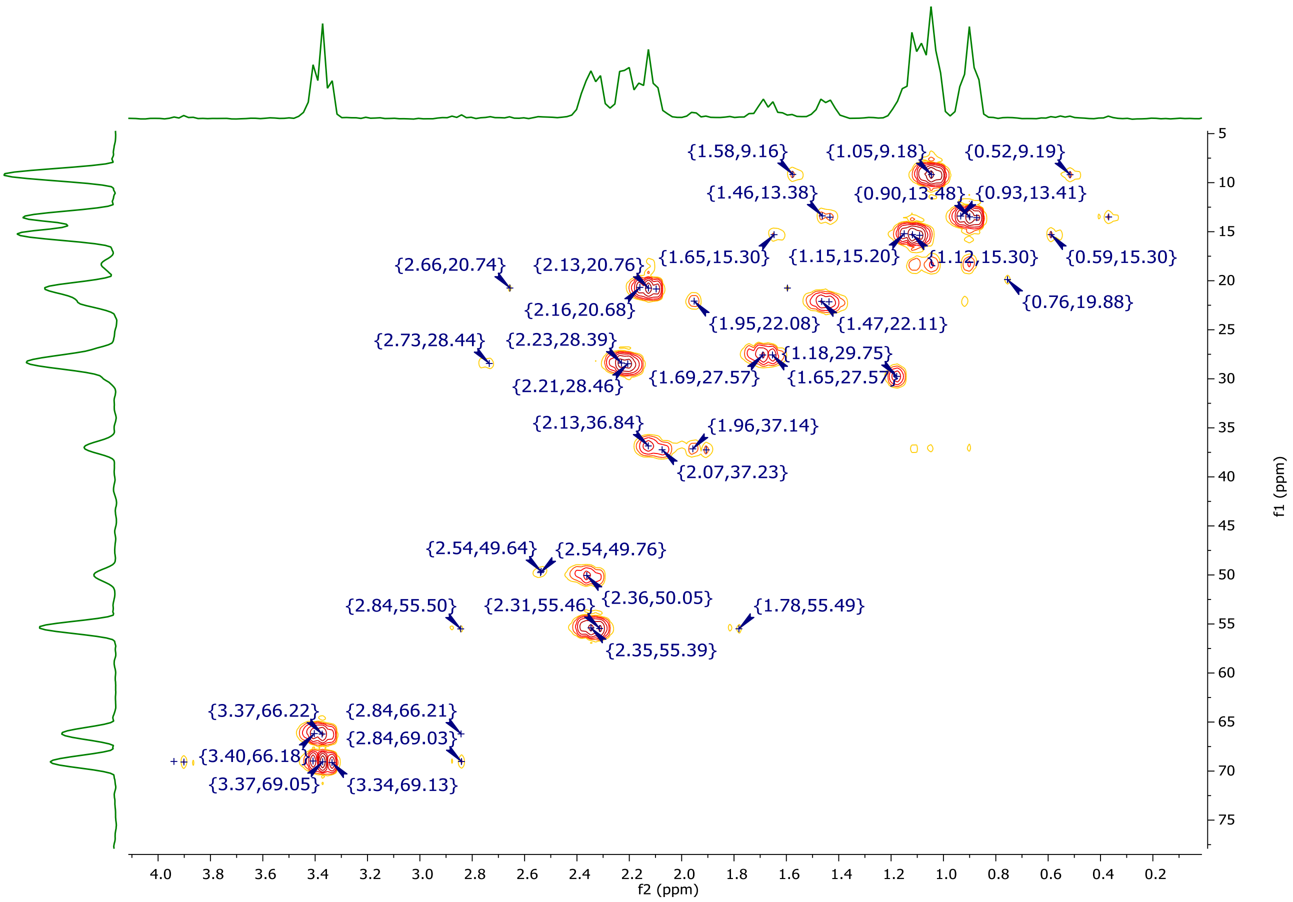

Figure S15. The HMQC spectrum of **EPP-pr**

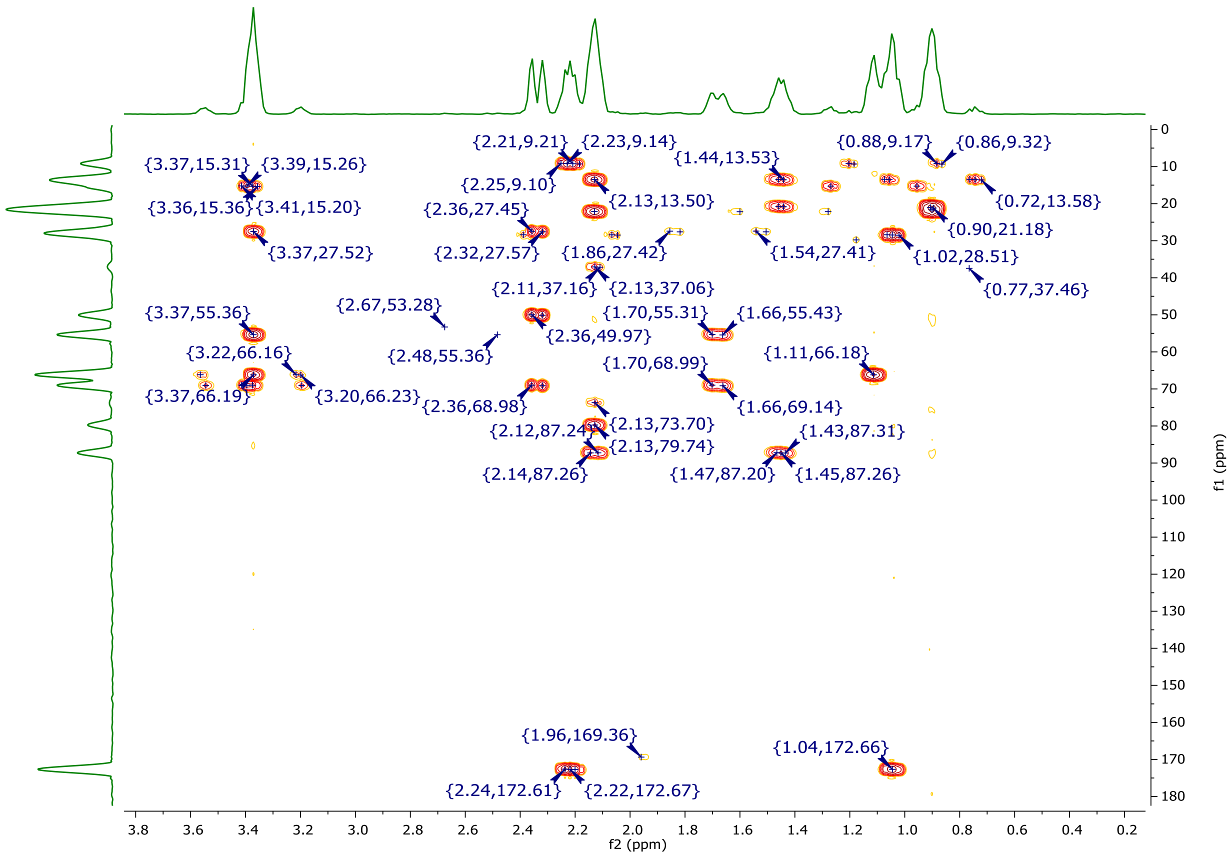

Figure S16. The HMBC spectrum of **EPP-pr**

Analysis of the NMR spectra of EPP-pr in $CDCl_3$ and its complex with β-CD (MXF-2) in DMSO-d_6

In the ^{1}H NMR spectrum of the **EPP-pr – β-CD complex (MXF-2)**, the methyl protons H-20,20,20 of the pentenyl fragment appear as a three-proton multiplet at 0.88–0.91 ppm. The methylene protons H-19,19 are observed as a two-proton multiplet at 1.35–1.44 ppm, while the remaining methylene protons H-18,18 resonate as a two-proton multiplet at 2.12–2.16 ppm. The piperidine protons H-3ax,5ax and H-3eq,5eq appear as two two-proton multiplets at 1.85–1.89 and 1.98–2.01 ppm, respectively.The methyl protons H-22,22,22 of the propionyloxy group give a three-proton multiplet at 0.94–0.98 ppm. The methylene protons H-21,21 resonate together with the methylene protons H-7,7 of the N-ethoxypropyl fragment and with the piperidine protons H-2ax,6ax as a six-proton multiplet at 2.19–2.29 ppm. The methyl protons H-12,12,12 of the N-ethoxypropyl fragment give a three-proton multiplet at 1.03–1.06 ppm, whereas the methylene protons H-8,8 appear as a two-proton multiplet at 1.54–1.61 ppm. The piperidine protons H-2eq,6eq are registered together with the solvent signal as a singlet at 2.47 ppm. The remaining methylene protons H-11,11 and H-9,9 of the N-ethoxypropyl fragment resonate as a multiplet at 3.28–3.32 ppm.

In the spectrum **MXF-22**, the oligosaccharide protons H-2 and H-4 appear as multiplets at 3.23–3.25 and 3.30–3.32 ppm, respectively. The β-cyclodextrin protons H-5, H-3, and H-6 are observed as multiplet signals at 3.50–3.52, 3.52–3.54, and 3.57–3.59 ppm. The anomeric proton H-1 is registered as a singlet at 4.78 ppm.

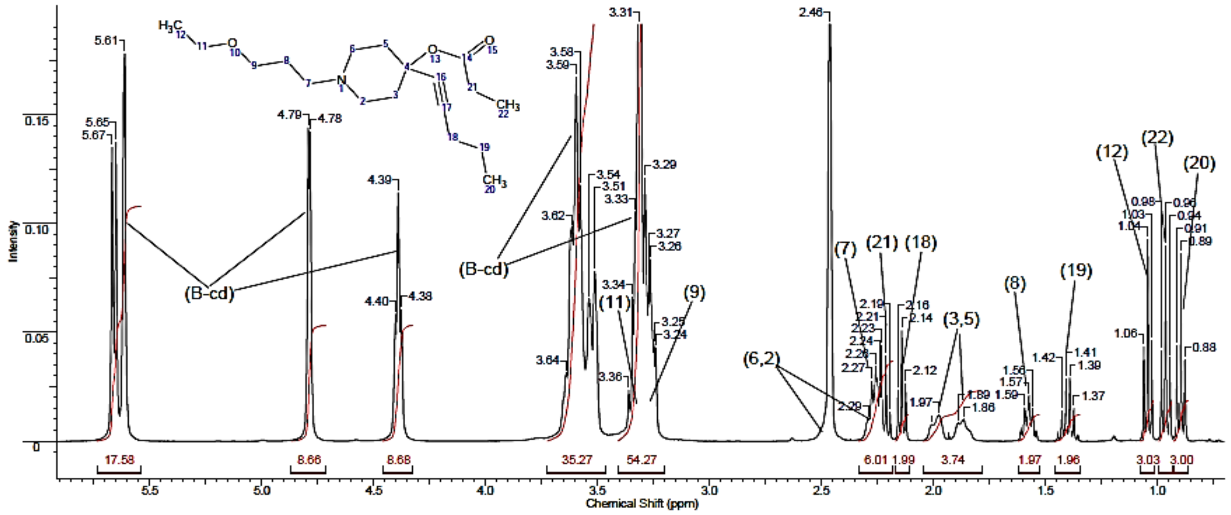

Figure S17. The ^{1}H NMR spectrum of **MXF-2 in DMSO-d_6**

In the ^{13}C NMR spectrum of the **MXF-22** complex, the carbon atoms of the pentenyl fragment resonate at 13.65 (C-20), 20.44 (C-18), 22.06 (C-19), 80.81 (C-16), and 87.29 (C-17) ppm. The carbons of the N-ethoxypropyl fragment appear at 15.54 (C-12), 27.48 (C-8), 55.14 (C-7), 65.74 (C-11), and 68.58 (C-9) ppm. The piperidine ring carbons are observed at 37.14 (C-3,5), 49.92 (C-2,6), and 73.70 (C-4) ppm. The propionyloxy group carbons resonate at 9.61 (C-22), 28.19 (C-21), and 172.13 (C-14) ppm.

In the ^{13}C NMR spectrum of **MXF-22**, the oligosaccharide carbons appear at 60.46 (C-6), 72.57 (C-5), 72.95 (C-2), 73.70 (C-3), 82.09 (C-4), and 102.32 (C-1) ppm.

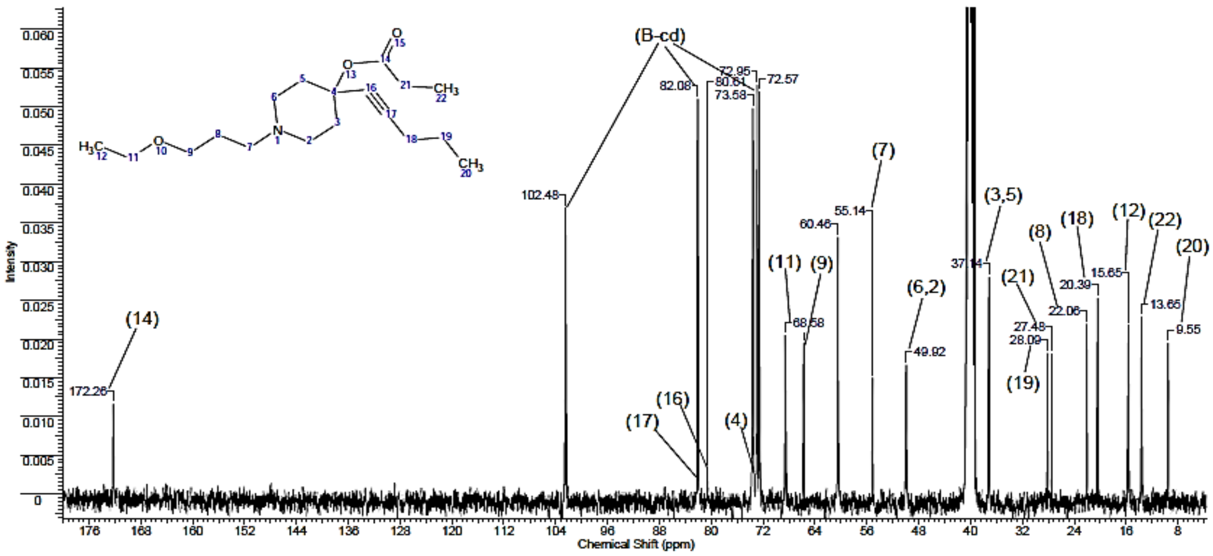

Figure S18. The ^{13}C NMR spectrum of **MXF-2 in DMSO-d6**

The structure of the **MXF-22** complex was further confirmed by 2D NMR experiments, including COSY (^{1}H–^{1}H), HMQC (^{1}H–^{13}C), and HMBC (^{1}H–^{13}C), which established both homo- and heteronuclear spin–spin correlations.

In the ^{1}H–^{1}H COSY spectrum of **MXF-22**, three-bond correlations were observed between neighboring methyl–methylene and methylene–methylene protons of the **EPP-pr** fragment: H20→H19 (0.88, 1.38), H22→H21 (0.94, 2.19), H19→H18 (1.39, 2.12), H12→H11 (1.02, 3.31), H8→H9 (1.55, 3.29), and H8→H7 (1.55, 2.25).

Three-bond correlations were also detected for the oligosaccharide moiety: H4→H3 (3.29, 3.53) and H2→H1 (3.25, 4.77).

One-bond heteronuclear correlations in the **MXF-22** complex (HMQC) were observed for the following EPP-pr pairs: H22→C22 (0.95, 9.57), H20→C20 (0.88, 13.72), H18→C18 (2.12, 20.45), H12→C12 (1.02, 15.70), H19→C19 (1.39, 22.09), H21→C21 (2.21, 28.04), H8→C8 (1.55, 27.53), H7→C7 (2.25, 55.06), H11→C11 (3.31, 65.82), H9→C9 (3.29, 68.26), H3ax,5ax→C3,5 (1.84, 37.28), H3eq,5eq→C3,5 (2.00, 37.02), H2ax,6ax→C2,6 (2.28, 49.97), and H2eq,6eq→C2,6 (2.48, 49.97).

HMQC correlations were also established for oligosaccharide carbons: H1→C1 (4.77, 102.63), H2→C2 (3.24, 73.04), H3→C3 (3.53, 73.70), H4→C4 (3.29, 82.10), H5→C5 (3.51, 72.77), and H6→C6 (3.58, 60.58).

Long-range ^{1}H–^{13}C HMBC correlations for the **EPP-pr** fragment of **MXF-22** included: H20→C19 (0.88, 21.36); H22→C21, C14 (0.94, 28.63; 172.43); H12→C11 (1.02, 66.01); H19→C20, C17 (1.38, 13.84; 87.29); H18→C20, C19, C16, C17 (2.12, 13.84; 22.66; 80.81; 87.29).

For the oligosaccharide fragment, HMBC correlations were observed for: H4→C6, C1 (3.28, 60.33; 102.46), H1→C5, C3, C4 (4.77, 72.07; 74.32; 82.04).

More long-range intramolecular interactions observed in the NOESY (**Figure S22**), ROESY (**Figure S23**), and TOCSY (**Figure S24**) spectra of **MXF-22** further support the formation of the inclusion complex.

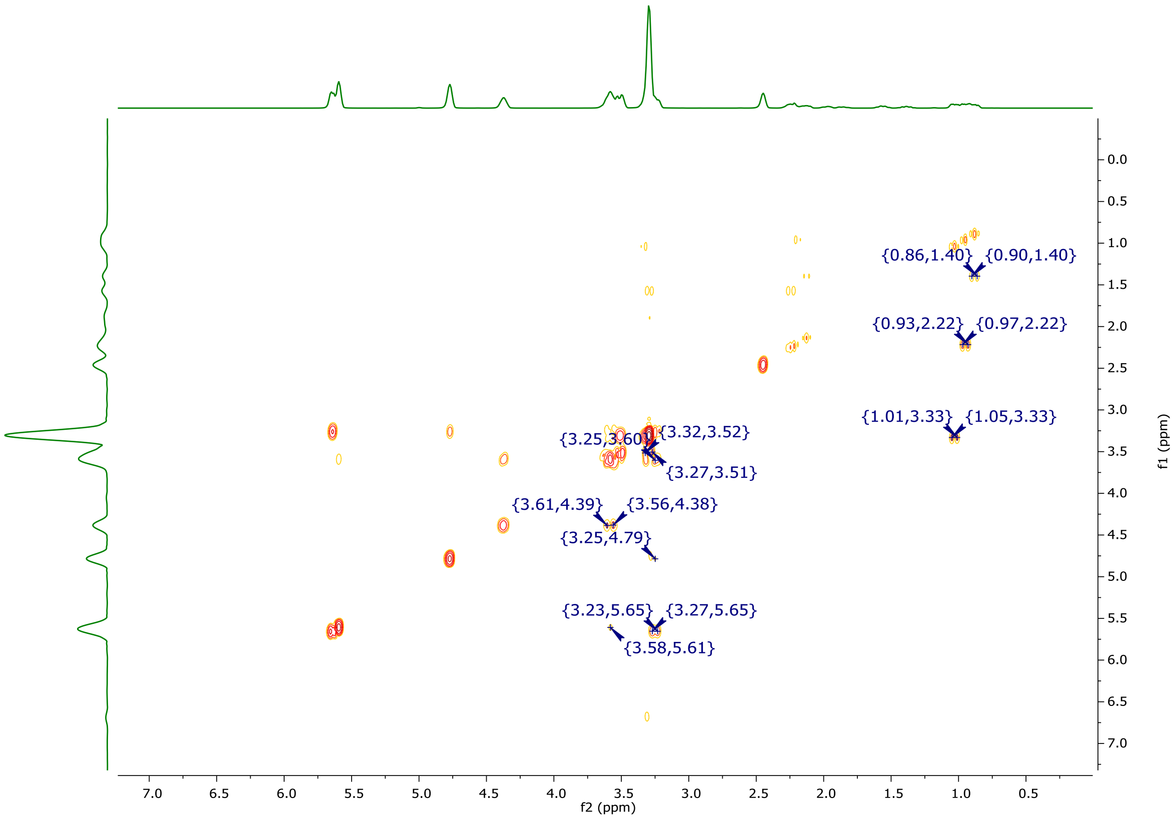

Figure S19. The COSY spectrum of **MXF-22**

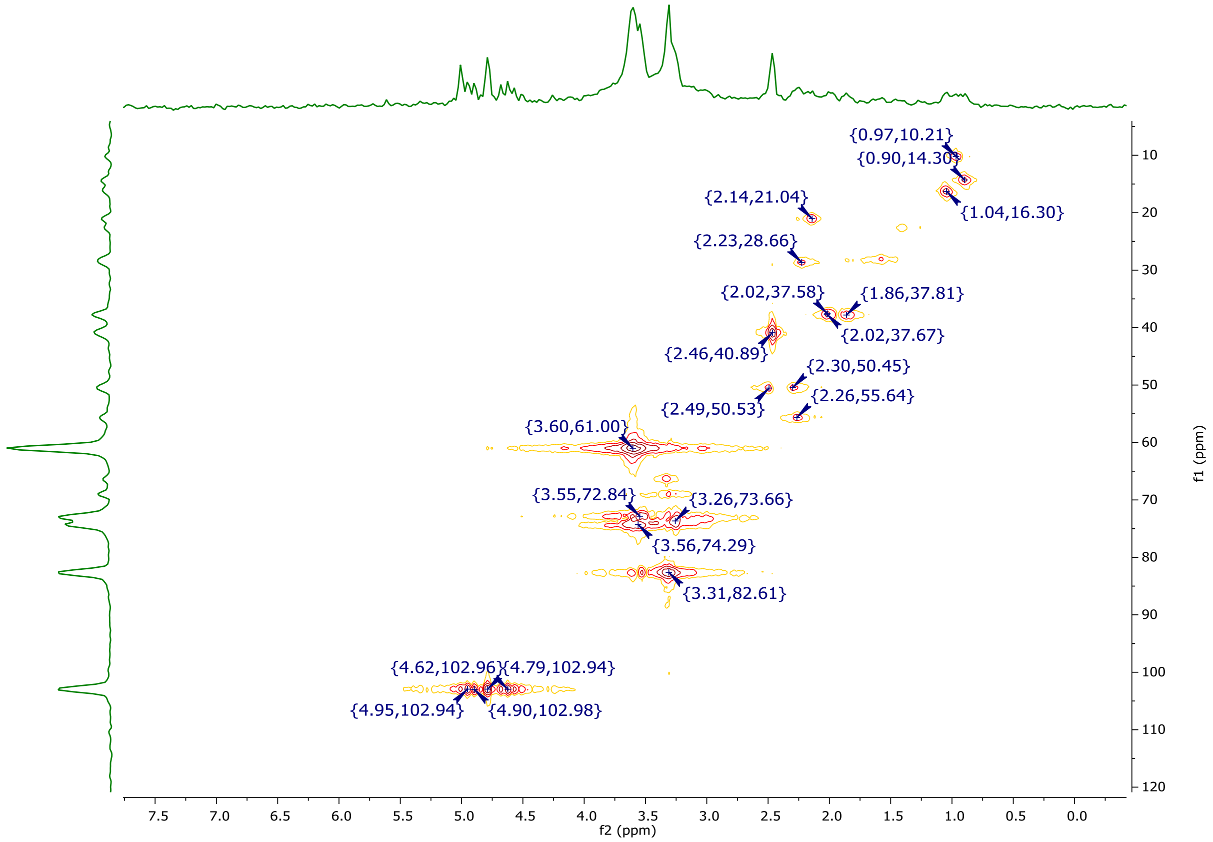

Figure S 20. The HMQC spectrum of **MXF-22**

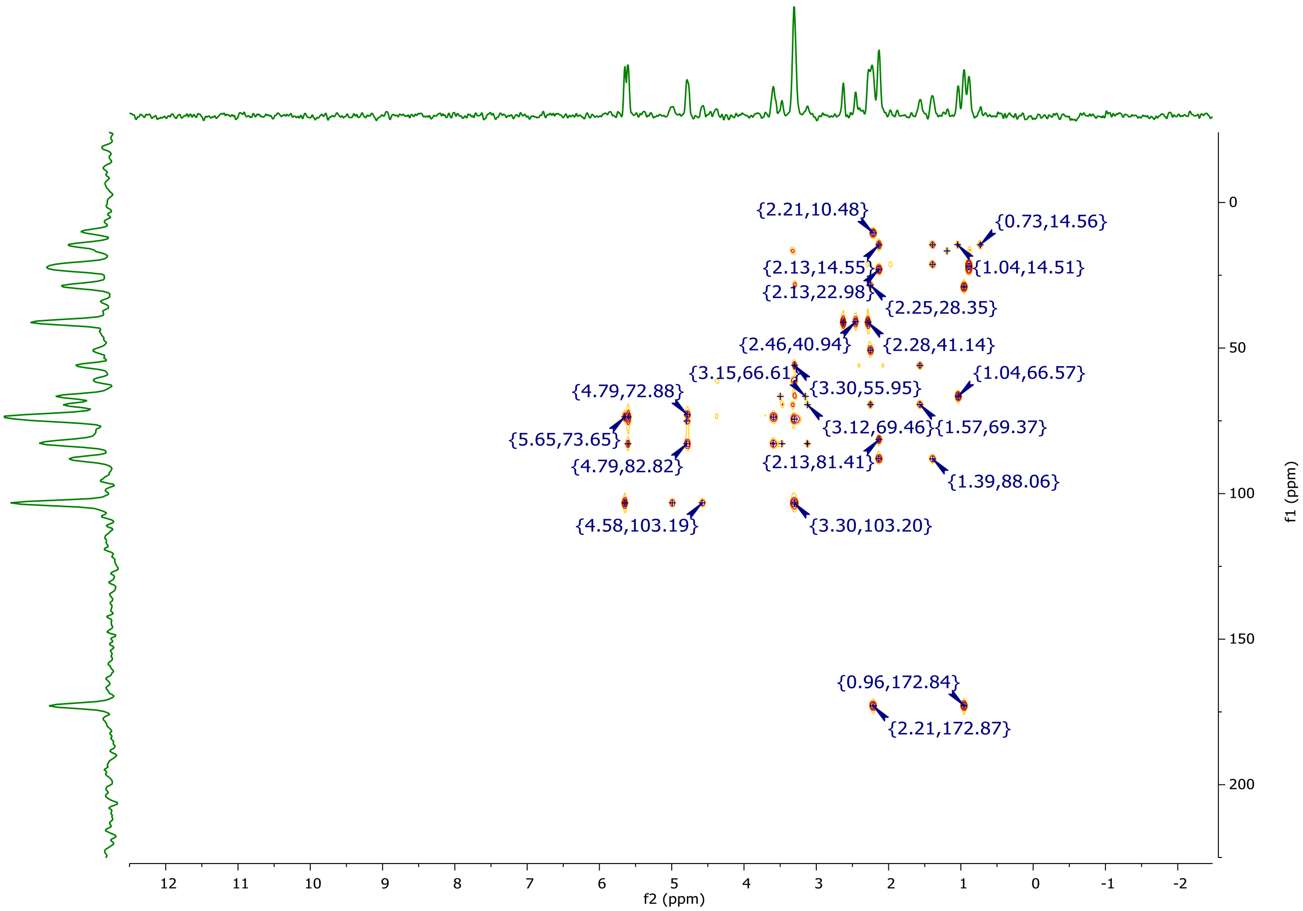

Figure S 21. The HMBC spectrum of **MXF-22**

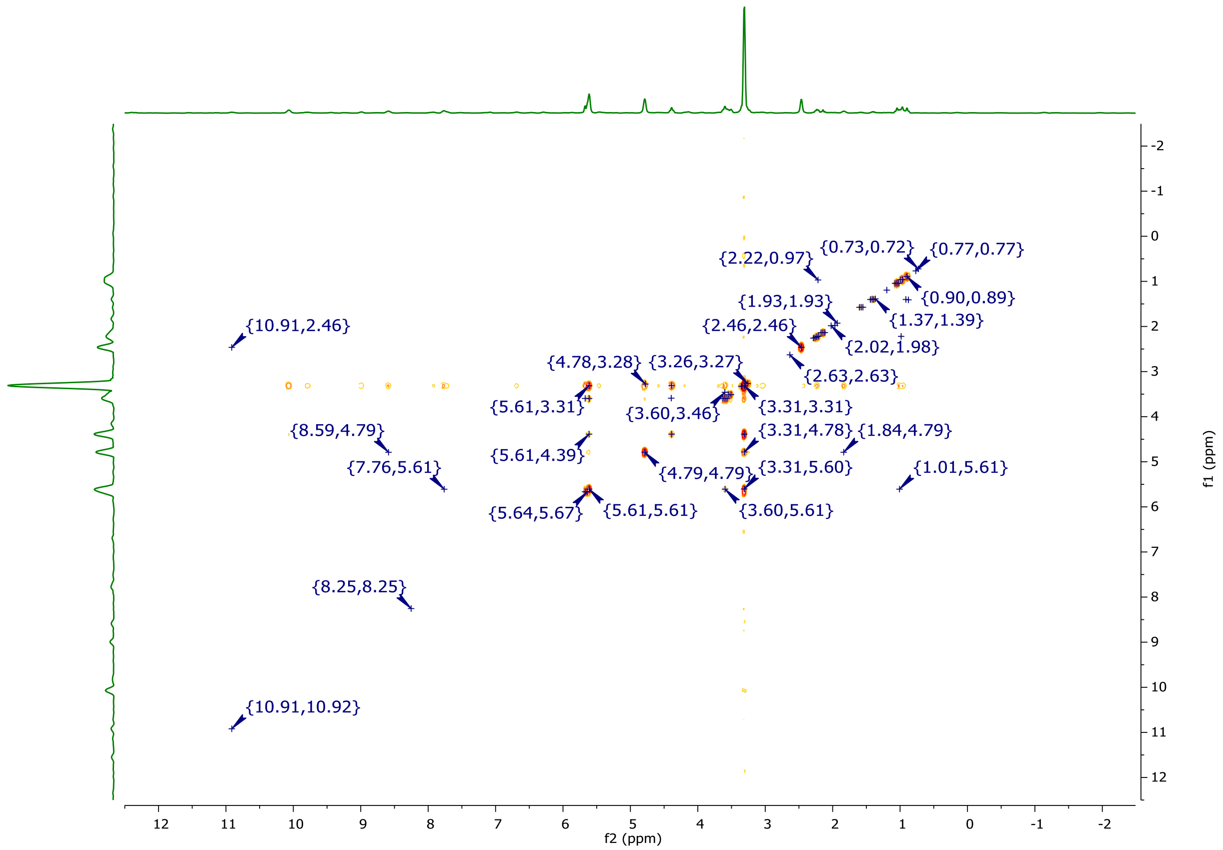

Figure S 22. The NOESY spectrum of **MXF-22**

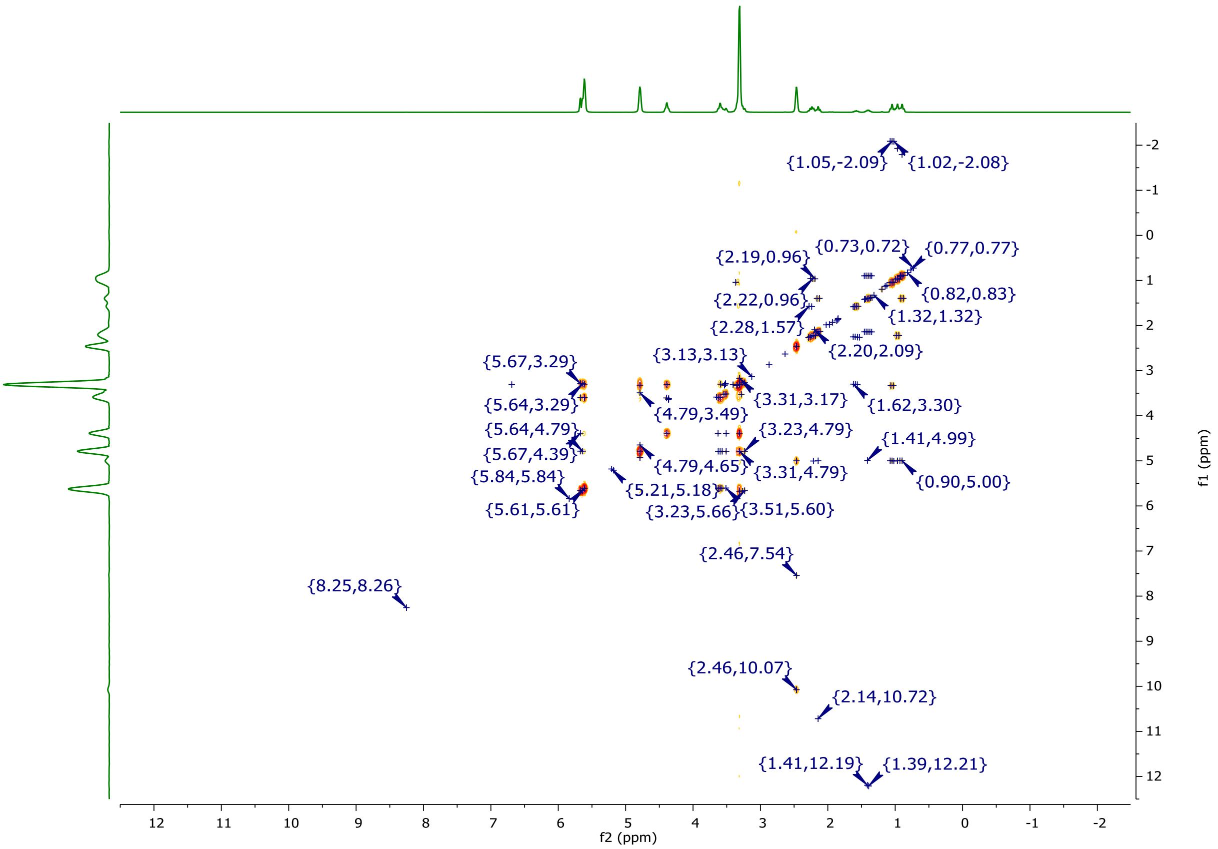

Figure S 23. The ROESY spectrum of **MXF-22**

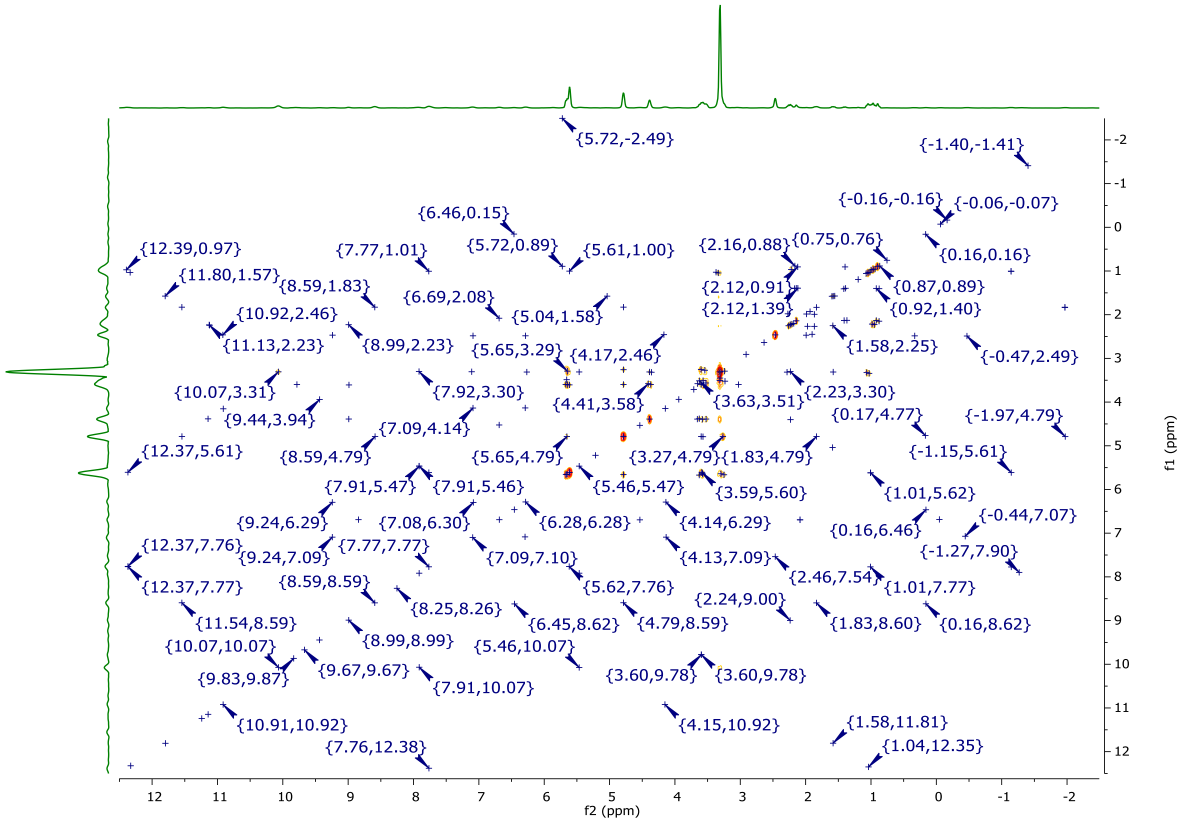

Figure S 24. The TOCSY spectrum of **MXF-22**

Shifts in chemical shifts in the β-CD proton spectrum (**Table S1**) occurred for protons directed inside the cyclodextrin cavity (H-3: Δδ = –0.02 ppm; H-5: Δδ = +0.02 ppm) as well as for protons on the outer surface (H-1: Δδ = +0.02 ppm; H-2: Δδ = –0.01 ppm; H-4: Δδ = +0.03 ppm;

H-6: Δδ = +0.02 ppm). Both shielding and deshielding effects were observed, supporting the formation of mixed supramolecular complexes **MXF-22**.

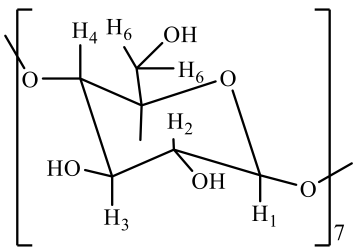

Figure S25. Some Numbered Protons of the β-CD Glucopyranose Moiety

Таблица S1. Chemical shifts of the ^{1}H and ^{13}C nuclei of **EPP-pr** and β-cyclodextrin in their free states (δ_0) and in the inclusion complex - **MXF-22** (δ).

Atom #	Group	δ_0, ppm, $CDCl_3$		δ, ppm, DMSO-d_6		$\Delta\delta = \delta - \delta_0$	
	CH_x	^{1}H	^{13}C	^{1}H	^{13}C	^{1}H	^{13}C
**EPP-pr**							
2ax,6ax	CH_2	2.33-2.39 m	50.04	2.27-2.29 m	49.92	-0.08	-0.48
2eq,6eq		2.52 br. s		2.47-2.49 m		-0.04	
3ax,5ax	CH_2	1.97 br. s	37.02	1.85-1.89 m	37.14	-0.10	0.12
3eq,5eq		2.10-2.16 m		1.98-2.01 m		-0.14	
4	>C<	-	73.70	-	73.70	-	0
7	CH_2	2.33-2.39 m	55.35	2.24-2.26 m	55.14	-0.11	-0.21
8	CH_2	1.66-1.73 m	27.50	1.54-1.61 m	27.48	-0.12	-0.02
9	CH_2	3.37-3.43 m	69.05	3.30-3.32 m	68.58	0.09	-0.47
11	CH_2	3.37-3.43 m	66.16	3.28-3.30 m	65.74	0.09	-0.42
12	CH_3	1.11-1.15 m	15.27	1.03-1.06 m	15.54	-0.09	0.27
14	-COO-	-	172.77	-	172.13		-0.64
16	-C≡	-	79.72	-	80.81		1.09
17	-C≡	-	87.22	-	87.29		0.07
18	CH_2	2.10-2.16 m	20.57	2.12-2.16 m	20.44	0.01	-0.3
19	CH_2	1.42-1.51 m	22.13	1.35-1.44 m	22.06	-0.07	-0.06
20	CH_3	0.88-0.93 m	13.48	0.88-0.91 m	13.65	-0.01	0.17
21	CH_2	2.21-2.26 m	28.60	2.20-2.22 m	28.19	-0.02	-0.41
22	CH_3	1.04-1.08 m	9.16	0.94-0.98 m	9.61	-0.10	0.45
**β-CD,** DMSO-d_6							
1	>CH	4.76 s	102.42	4.78 s	102.32	0.02	-0.10

2	>CH	3.22-3.27 m	72.86	3.23-3.25 m	72.95	-0.01	0.09
3	>CH	3.48-3.55 m	73.54	3.52-3.54 m	73.70	-0.02	0.16
4	>CH	3.27-3.29 m	82.01	3.30-3.32 m	82.09	0.03	0.08
5	>CH	3.49 s	72.51	3.50-3.52 m	72.57	0.02	0.06
6	CH_2	3.55-3.57 m	60.42	3.57-3.59 m	60.46	0.02	0.04

Shifts in the β-CD proton spectrum (**Table S1**) were observed both for the protons oriented toward the cyclodextrin cavity (H-3: Δδ = –0.02 ppm; H-5: Δδ = +0.02 ppm) and for those located on the outer surface (H-1: Δδ = +0.02 ppm; H-2: Δδ = –0.01 ppm; H-4: Δδ = +0.03 ppm; H-6: Δδ = +0.02 ppm). The combination of shielding and deshielding effects is consistent with the formation of mixed supramolecular **MXF-22** complexes.

During supramolecular binding of **EPP-pr** with β-CD oligomers, the most pronounced changes in chemical shifts were detected for the piperidine protons H-3eq,5eq (Δδ = –0.14 ppm) and H-3ax,5ax (Δδ = –0.10 ppm), as well as for several methylene groups of the N-ethoxypropyl substituent: H-7,7 (Δδ = –0.11 ppm), H-8,8 (Δδ = –0.12 ppm), H-9,9 (Δδ = +0.09 ppm), H-11,11 (Δδ = +0.09 ppm), and the methyl protons H-12,12,12 (Δδ = –0.09 ppm). Notable changes were also observed for the methyl group of the propionyloxy moiety H-20,20,20 (Δδ = –0.10 ppm). These data indicate that the protons showing the largest deviations in chemical shift are likely directly involved in supramolecular interactions with the glucopyranose residues of β-CD.

Analysis of the integral ratios revealed that in the **MXF-22** inclusion complex, approximately one β-cyclodextrin molecule is associated with one molecule of **EPP-pr**.
